# Fully co-factor-free ClearTau platform produces seeding-competent Tau fibrils for reconstructing pathological Tau aggregates

**DOI:** 10.1038/s41467-023-39314-7

**Published:** 2023-07-04

**Authors:** Galina Limorenko, Meltem Tatli, Rajasekhar Kolla, Sergey Nazarov, Marie-Theres Weil, David C. Schöndorf, Daniela Geist, Peter Reinhardt, Dagmar E. Ehrnhoefer, Henning Stahlberg, Laura Gasparini, Hilal A. Lashuel

**Affiliations:** 1 Laboratory of Molecular and Chemical Biology of Neurodegeneration, Institute of Bioengineering, School of Life Sciences, Ecole Polytechnique Fédérale de Lausanne, CH-1015 Lausanne, Switzerland; 2 Laboratory of Biological Electron Microscopy, Institute of Physics, School of Basic Sciences, Ecole Polytechnique Fédérale de Lausanne, CH-1015 Lausanne, Switzerland; 3 Biological Electron Microscopy Facility, School of Life Sciences, Ecole Polytechnique Fédérale de Lausanne, CH-1015 Lausanne, Switzerland; 4 Neuroscience Discovery, AbbVie Deutschland GmbH & Co KG, Knollstrasse, 67061 Ludwigshafen, Germany; 5 Department of Fund. Microbiology, Faculty of Biology and Medicine, University of Lausanne, CH-1015 Lausanne, Switzerland

**Keywords:** Assay systems, Protein aggregation

## Abstract

Tau protein fibrillization is implicated in the pathogenesis of several neurodegenerative diseases collectively known as Tauopathies. For decades, investigating Tau fibrillization in vitro has required the addition of polyanions or other co-factors to induce its misfolding and aggregation, with heparin being the most commonly used. However, heparin-induced Tau fibrils exhibit high morphological heterogeneity and a striking structural divergence from Tau fibrils isolated from Tauopathies patients’ brains at ultra- and macro-structural levels. To address these limitations, we developed a quick, cheap, and effective method for producing completely co-factor-free fibrils from all full-length Tau isoforms and mixtures thereof. We show that Tau fibrils generated using this ClearTau method – ClearTau fibrils - exhibit amyloid-like features, possess seeding activity in biosensor cells and hiPSC-derived neurons, retain RNA-binding capacity, and have morphological properties and structures more reminiscent of the properties of the brain-derived Tau fibrils. We present the proof-of-concept implementation of the ClearTau platform for screening Tau aggregation-modifying compounds. We demonstrate that these advances open opportunities to investigate the pathophysiology of disease-relevant Tau aggregates and will facilitate the development of Tau pathology-targeting and modifying therapies and PET tracers that can distinguish between different Tauopathies.

## Abstract (web summary)

The authors report a method for producing co-factor-free fibrils from all full-length Tau isoforms. The method paves the way for reconstituting pathology resembling Tau fibrils and enables screening of Tau aggregation-modifying compounds for targeted therapies and PET tracers.

## Introduction

The microtubule-binding protein (MAP) Tau is an intrinsically disordered protein (IDP) that is most prominently associated with the dynamic regulation and stabilization of cytoskeletal and mitotic microtubules^1^. In neurons, Tau is also important for regulating axon outgrowth and maintaining axonal transport and cytoskeletal integrity^2^. However, factors such as post-translational modifications (PTMs)^3^, mutations in the protein sequence^4^, interaction with other proteins^5,6^ and changes to the biochemistry of its surrounding environment, such as pH or the presence of drugs^7^, may result in the lowered affinity, weaker interaction, or full dissociation of Tau from microtubules (for a recent review see^8^). Tau may then accumulate and aggregate into higher molecular weight species, such as fibrils associated with pathology. Increasing evidence points to Tau aggregation and PTMs as central events in the pathogenesis of Alzheimer’s disease (AD) and Tauopathies, events that investigators strive to faithfully model in the laboratory (for a recent review see^9^). In addition to amyloid plaques composed of β-amyloid, a classic hallmark of AD, another prominent pathological feature of AD is hyperphosphorylated Tau which is found in neuronal cell bodies or neurites in the form of paired helical filaments (PHFs) and straight filaments (SFs)^10^. Tau aggregates and fibrillar structures are also found in the brain of individuals afflicted by other neurodegenerative diseases (NDs), collectively known as Tauopathies, which include Pick’s disease (PiD) and progressive supranuclear palsy (PSP)^11–18^. Tau exists as six isoforms in the human central nervous system, designated 4R2N, 4R1N, 4R0N, 3R2N, 3R1N, and 3R0N. Where the Tau isoform compositions of the Tau fibrils are known^13^, Tauopathies are classified into predominantly 3 R (i.e., PiD), predominantly 4 R (i.e, PSP), or mixed (3 R + 4 R; i.e., AD).

Full-length Tau isoforms are highly soluble and notoriously resistant to aggregation on their own, in contrast to other amyloid-forming proteins, such as α-synuclein^19^, β-amyloid peptide^20^ or amylin^21^, which misfold, aggregate, and form amyloid fibrils simply by incubation at 37 °C. The molecular and cellular factors that trigger Tau misfolding and aggregation, and drive the Tau fibrillization processes remain unclear. Therefore, to study Tau fibrillization in vitro, investigators have adopted Tau aggregation systems using various negatively-charged co-factor molecules or protein modifications, such as truncations or phosphorylation, which are used to induce or accelerate the full-length Tau fibrillization process. Negatively-charged polysaccharide free-floating heparin (FFH) has thus far been the most commonly used Tau aggregation co-factor, although others including RNA, anionic lipids, or small proteins are occasionally used (reviewed in^22^). The mechanisms by which anionic co-factors induce Tau aggregation are thought to include electrostatic charge neutralization of highly positively-charged regions on Tau, promoting changes in the local and global Tau polypeptide shape^22^. This allows the folding of the aggregation-promoting Tau regions PHF6* and PHF6 into β-sheet-containing conformation, leading to Tau fibrillization and assembly into higher-order Tau fibrils. In addition, structural and biophysical studies have shown a high affinity of specific positively-charged Tau residues for heparin, notably the interfibrillar interface-forming residues in the core of the fibril folds.

**Fig. 1:**
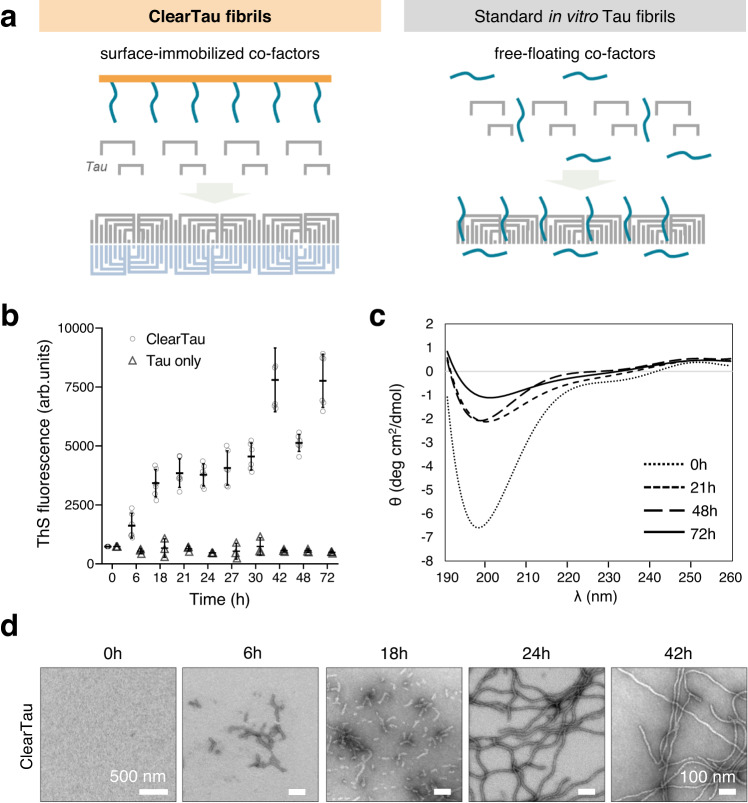
Characterization of ClearTau 4R2N Tau fibrils. **a** Schematic of the ClearTau and FFH-induced Tau fibrillization. In the ClearTau method, the heparin molecules are covalently immobilized on the aggregation vessel wall. The addition of Tau monomers up to the minimal composition buffer results in the rapid formation of the Tau fibrils from all six Tau isoforms composed of doublet filaments. In the presence of FFH, the association of heparin with Tau filaments may interfere with the doublet fibril formation. **b** ThS aggregation assay followed two independent ClearTau aggregation reactions, compared to the normal Eppendorf tube without additional co-factors. The ThS fluorescence signal is rapidly generated only for ClearTau fibrils over the course of 48 h and follows a sigmoidal curve reaching the plateau phase. All reactions comprised 10 μM of Tau monomer at the inception. Measurements were performed in triplicates. Data are presented as mean values +/− SD with all individual values plotted. **c** Circular dichroism (CD) spectra of ClearTau fibrils. **d** Electron microscopy imaging reveals the formation of the fibrils by 4R2N Tau in ClearTau aggregation reaction, with shorter fibrils appearing at 6 and 18 h, to long fibrils at day 1 and 2 of aggregation. Scale bars = 100 nm. For a wider profile of fibril morphologies, see Supplementary Fig. 1. The experiment was repeated for a minimum of three independent times with consistent results. Source data are provided as a Source Data file.

To be clinically relevant, in vitro Tau aggregation systems must recapitulate some of the morphological, biochemical, and structural features of Tau aggregates and fibrils found in human pathologies. Several high-resolution structures of Tau fibrils composed of different Tau isoforms have been recently solved using cryoelectron microscopy (cryo-EM; see^23^), including Tau filaments isolated from post-mortem patient brain tissues. These structures combined with other experimental observations suggest that heparin or other co-factors used to induce Tau aggregation in vitro introduce several limitations that preclude biologically-relevant studies from elucidating the role of Tau aggregation in the pathogenesis of Tauopathies (Fig. 1). First, recent studies demonstrated that in vitro FFH-induced Tau fibrils differ from pathological patient-derived fibrils at the biochemical (lack of PTM patterns due to the use of the unmodified isoforms) and structural (amyloid core conformation) levels^24^. In addition, the mature Tau fibrils in AD, corticobasal degeneration (CBD), and chronic traumatic encephalopathy (CTE) are composed of doublet Tau filaments with the interfilament interfaces differing between these disorders, which manifests in various twisted morphologies of the higher-order Tau fibrils. In contrast, conventional in vitro-produced Tau fibrils extensively comprise a single-filament and are structurally and morphologically divergent from any Tau fibrillar structures derived from ND patients’ brains, recently illustrated by cryo-EM-solved FFH-induced fibril structures^24^. Second, the addition of heparin to Tau results in heterogeneous fibril populations, whereas the ultrastructures of pathological Tau fibrils are consistent between patients and are a signatory of the specific Tauopathies. Third, heparin and other co-factors have been shown to bind strongly and remain associated with the Tau fibril, which could interfere with their interaction with other small molecules and ligands (e.g., RNAs, small-molecule compounds)^25,26^. The Tau fibrils induced by RNA also show no similarity to any known pathological Tau fibril structures^27^. These properties preclude the use of FFH- and RNA-induced Tau fibrils to investigate the structural basis of Tau aggregation, cellular uptake, and toxicity or their use for the development and testing of Tau-targeting and binding molecules, as well as positron emission tomography (PET) tracers.

To address these limitations, we developed an in vitro protein fibrillization method to generate co-factor-free Tau fibrils, ClearTau fibrils. This method uses surface-immobilized heparin to induce Tau misfolding and aggregation while preventing its tight association with and incorporation into the fibrils^25^ (Fig. 1a). Prior knowledge of the mechanisms involved in heparin-induced Tau fibrillization suggests that heparin likely has its principal contribution at the very early stages of the Tau fibrillization process. The binding of negatively-charged heparin to Tau neutralizes the positive charge on the Tau monomer and, importantly, on the aggregation-driving regions within the microtubule-binding region (MTBR), which contributes to the high solvability of Tau under normal conditions. This leads to misfolding of Tau through the loss of the long-range contacts between the N-terminus and central regions, as well as local restructuring of the MTBR to aggregation-prone configuration. After the heparin-dependent priming of the Tau monomers and assembly of the seeding-competent Tau nucleus, the self-assembly and further polymerization are thought to be Tau seed template-dependent and may not be reliant on the binding of the exogenous molecules such as heparin to the growing fibrils (for a recent review see^22^).

Therefore, we hypothesized that surface covalent immobilization of heparin would allow us to leverage the first and crucial step of the heparin action on Tau monomers, at the same time completely preventing its incorporation into the growing Tau fibrils as the heparin molecules are bound to the vessel surface, whereas Tau molecules are free-floating in the large volume of the aqueous buffer (Fig. 1a). To test this hypothesis, we assessed the efficiency of fibrillization of all six Tau isoforms, mixtures of isoforms, mutant and Tau fragments in Eppendorf tubes coated with covalently-immobilized heparin, i.e., in the absence of FFH. Our results show that this method allows for the efficient production of a large amount of homogeneously-aggregated co-factor-free fibrils that are morphologically distinct from those produced in the presence of FFH. The ClearTau fibrils retain the ability to bind RNA, share some structural/morphological features with brain-derived pathological fibrils, and induce efficient seeding of Tau aggregation in biosensor cells and human induced pluripotent stem cell (hiPSC)-derived neurons. Our method for in vitro ClearTau fibril production represents a crucial milestone for developing accessible, cheap, and high-fidelity tools for studying the Tau fibrillization processes relevant to NDs. As a proof of concept, we demonstrate that the ClearTau method can be used as a platform to screen small molecule-based modulators of Tau aggregation and to identify the stabilizers of heparin-free Tau oligomers. The ease of implementation, availability of the heparin-coating methods^28^, and minimal buffer composition requirements (high efficiency in the phosphate-buffered saline, PBS and H_2_O only) allow for the production of high yields of clean co-factor-free fibrils for in vitro and in vivo mechanistic studies and for drug screenings aimed at developing efficient diagnostic tools and therapies to treat AD and other Tauopathies. The generation of ClearTau fibrils also paves the way for a more systematic analysis of Tau post-fibrillization PTMs and fibril interactome without interference from heparin or heparin-induced polymorphisms. Finally, we provide a platform concept for high-throughput studies of pathology-relevant Tau aggregation for drug development.

## Results

### ClearTau method for generating co-factor-free fibrils

To determine if the surface-immobilized heparin retains the ability to induce Tau fibrillization, we assessed and compared the extent of Tau 4R2N monomers (10 μM) fibril formation in 1.5 ml Eppendorf tubes that are covalently coated with heparin molecules and in standard 1.5 ml Eppendorf tubes containing no FFH. The kinetics of aggregation was followed by the thioflavin S fluorescence (ThS) assay over the course of 72 h. As shown in Fig. 1b, a rapid increase in ThS fluorescence was only observed in the heparin-coated tubes, whereas no change in ThS signal was detected in the standard Eppendorf tube without immobilized heparin or FFH at this concentration. Circular dichroism (CD) measurement of the samples during the reaction revealed a significant drop in the CD signal, suggesting that the majority of the Tau monomer has converted to insoluble Tau fibrils that precipitate out of solution as early as 1 day into the reaction progression (Fig. 1c). Electron microscopy (EM) imaging revealed the formation of short fibrillar aggregates at 6 and 18 h into the reaction time course, with the dominant appearance of long curvy fibrils at 24 h and later (Fig. 1d, Supplementary Fig. 1). These results demonstrate that our ClearTau method is efficient for the fibrillization of Tau, is easy to implement, and is highly versatile.

### ClearTau method induces efficient fibrillization of all six isoforms, mutant, and fragments of Tau into fibrils different from FFH-induced Tau fibrils

**Fig. 2:**
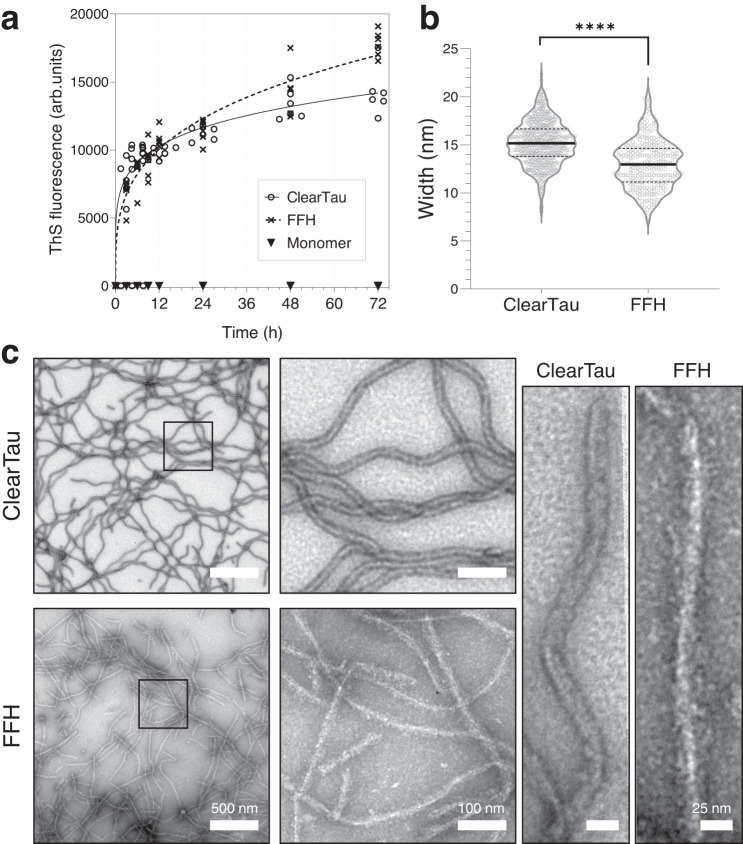
Comparison of 4R2N Tau fibrils generated by ClearTau versus FFH methods. **a** ThS aggregation kinetics comparison of ClearTau and FFH-induced Tau fibrils. FFH was added to the 4R2N Tau monomer at 1:4 molar concentration both reactions comprised 100 μM of Tau monomers. Reactions were set up as four independent replicates, and measurements were performed in triplicates. Data are presented as all individual values plotted. **b** Width quantification of fibrils shows significantly narrower FFH fibrils. *N* of ClearTau = 618, N of FFH = 292 fibrils quantified. Two-way ANOVA, significant values are denoted by: ns *P* > 0.05, *P ≤ 0.05, **P ≤ 0.01, ***P ≤ 0.001, ****P ≤ 0.0001. Exact *p*-values can be found in the Source Data file. **c** Electron microscopy imaging reveals the formation of the fibrillar structures in both reactions over 72 h of differing morphologies. The experiment was repeated of minimum three independent times with consistent results. Scale bars = 500 and 100 nm. Scale bars = 25 nm. Source data are provided as a Source Data file.

Next, we compared the kinetics of aggregation of Tau 4R2N using our ClearTau method to the conventional FFH at a high concentration of 100 μM, which favors the formation of fibrils. As shown in Fig. 2a, a rapid increase in ThS fluorescence as early as 8 h was observed using both the ClearTau and FFH systems, illustrating the comparable kinetic profiles of Tau fibrillization at this concentration. EM imagining of the fibrils revealed the formation of the fibrils under both conditions. However, the fibril morphologies differed substantially (Fig. 2b, c). ClearTau fibrils were long, wavy, and of consistent morphology, with no accumulation of intermediate oligomers or amorphous aggregates. In contrast, FFH-induced Tau fibrils were fragmented and lacked consistent twisting. These results suggest that ClearTau fibrils could differ in their ultrastructure properties. Therefore, we measured and quantified the fibril widths of the ClearTau and FFH-induced Tau fibrils (Fig. 2b). The mean width of the ClearTau fibrils was greater than the FFH-induced fibrils (15.2 ± 2.24 nm and 13 ± 2.5 nm, respectively, p«0.001). We also assessed and compared aggregation of 4R2N, 4R0N, 3R2N, 3R0N, mutant 4R2N P301L, and fragments K18 and K19 using ClearTau or FFH systems. The EM imaging revealed consistently longer and more homogeneous fibrils in the ClearTau method (Supplementary Fig. 2) and illustrated the versatility of the ClearTau method’s use with a variety of Tau proteins and fragments. These results show that our ClearTau method for Tau fibrillization (1) works on experimentally-relevant time scales; (2) is devoid of Tau oligomers or amorphous aggregates; and (3) produces long fibrils with consistent morphology, rather than variably twisted or straight fibrils within a sample.

### ClearTau fibrils’ seeding potency in vitro and cells

To assess whether 4R2N ClearTau fibrils were competent to seed aggregation of the Tau monomer in the absence of any co-factors in vitro, the aggregation assay was performed using a microplate setup (Supplementary Fig. 3a). Tau 4R2N monomers were seeded with either sonicated 4R2N ClearTau or FFH fibrils. There was a rapid increase in the ThS fluorescence in the FFH fibril-seeded reaction right after the addition of the seed. However, the aggregation kinetics profiles of ClearTau-seeded samples did not differ from Tau monomer-only set up. These results suggest that co-factors in the cellular milieu or Tau PTMs play critical roles in regulating the seeding activity of full-length ClearTau. To test this hypothesis, we assessed the seeding capacity of ClearTau seeds in cellular systems. The formation of fluorescent foci in the HEK293T TauRD P301S biosensor (BS) cell line is commonly used to assess Tau seeding activity^29^ (reviewed in^30^). These BS cells stably express the repeat domain of Tau with the P301S mutation (TauRD P301S) fused with a yellow fluorescent protein (YFP) or cyan fluorescent protein (CFP). The FRET signal is generated upon the reporter foci formation in these cells, where the two fluorophores, YFP and CFP, come in close proximity for the wavelength energy transfer, the signal from which is then detected and quantified using a flow cytometry set up as initially described^31^. The results in Supplementary Fig. 3b show the FRET signal’s detection is contingent on the amount of the fibrillar seeds added, demonstrating the dose-dependency of the seeding with ClearTau fibrils in this cellular model. In addition, confocal imaging verified the presence of both cytoplasmic (Supplementary Fig. 3c, red arrows) and nuclear reporter foci (Supplementary Fig. 3c, yellow arrowheads), whereas none were present in the non-transduced cultures.

**Fig. 3:**
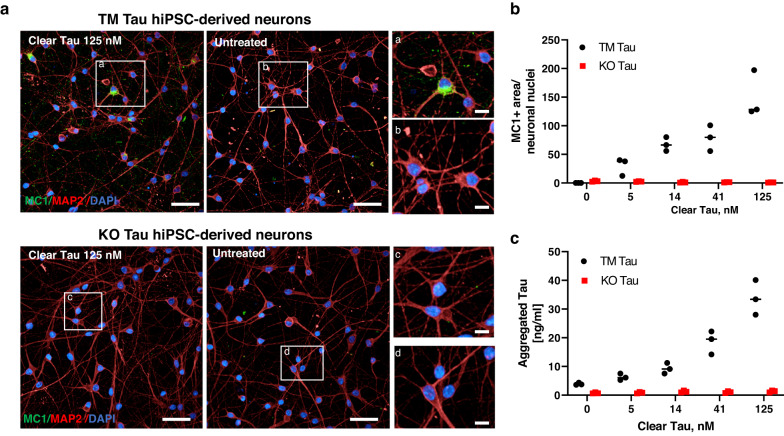
Clear Tau fibrils induced dose-dependent aggregation of endogenous Tau in TM Tau, but not KO Tau, hiPSC-derived cortical neurons. TM Tau and KO Tau hiPS-derived cortical neurons were exposed to different amounts of ClearTau fibrils generated from recombinant P301L 2N4R Tau. 3 weeks later, the neurons were stained with the MC1 Tau antibody to detect endogenous Tau aggregates (green) and MAP2 to stain the neurites (red). Nuclei were stained by DAPI. **a** Representative stack images. Scale bar 50 μm in main panels; 10 μm in insets (**a**–**d**). **b** Quantification of MC1 positive (MC1+) area over neuronal nuclei. Two-way ANOVA: seed concentration *p* < 0.0001, genotype *p* < 0.0001. **c** Aggregation of endogenous Tau was evaluated by symmetric ELISA using Tau12 antibody for both capture and detection. The graphs show results from one experiment with 3 replicates/condition. Two-way ANOVA: Tau concentration *p* < 0.0001, genotype *p* < 0.0001. Images and graphs represent data from one experiment. Two independent experiments with three replicates/condition were performed for immunofluorescence analyses. Source data are provided as a Source Data file.

Next, we investigated the ability of Clear Tau fibrils to seed the aggregation of endogenous Tau in neurons. ClearTau fibrils were applied to the hiPSC-derived neurons and aggregation of endogenous Tau was examined by immunofluorescence and biochemical analyses. The triple mutant Tau MAPT-P301S/E10 + 16 (TM) and knockout (KO) Tau hiPSC-derived cortical neurons were exposed to different amounts of ClearTau fibrils generated from recombinant P301L 2N4R Tau (See Methods and as previously described^32^). Three weeks later, the neurons were stained with the MC1 Tau antibody to detect endogenous Tau aggregates (Fig. 3a, green) and MAP2 to stain the neurites (Fig. 3a red). ClearTau fibrils induced the formation of Tau aggregates in TM Tau hiPSC-derived neurons in a concentration-dependent manner as demonstrated by the quantification of the MC1 signal (Fig. 3b) and symmetric ELISA using Tau12 antibody for both capture and detection (Fig. 3c). No Tau aggregates were detected when ClearTau fibrils were applied to KO Tau hiPSC-neurons, indicating that endogenous Tau expression is required for aggregates formation (Fig. 3). FFH Tau fibrils also induced aggregation of endogenous Tau in TM Tau hiPSC-derived neurons (Supplementary Fig. 4) as previously described^32^. These results demonstrate that ClearTau fibrils efficiently induce reporter foci formation in the HEK293T TauRD P301S biosensor cell line, and trigger endogenous Tau aggregation in TM Tau hiPSC-derived neurons, and the seeding is likely co-factor-dependent.

### Homogeneous ClearTau fibrils composed of singular Tau isoforms are structurally diverse between the isoforms

In human CNS, Tau is expressed as six isoforms differing in the number of incorporated N-terminal repeats, and differential inclusion of the R2 domain in the MTBR. As different isoforms contribute to Tau aggregation in the specific Tauopathies, it is important to develop high-fidelity methods for in vitro production of Tau fibrils of all six Tau isoforms or mixtures of the specific isoforms.

**Fig. 4:**
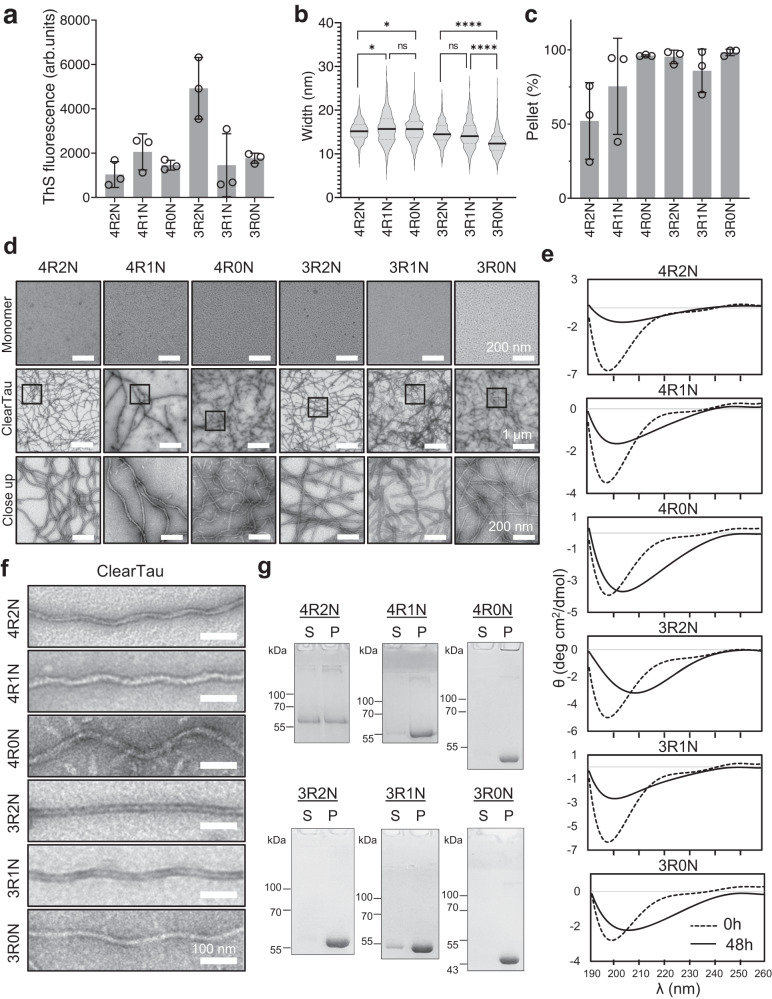
Characterization of ClearTau Tau fibrils generated from all six Tau isoforms. **a** Reaction endpoint at ThS fluorescence shows the signal for all isoforms, with the highest ThS signal found for ClearTau 3R2N isoforms, likely due to the unique straight fibril morphology. Three independent ClearTau aggregation reactions for each Tau isoform were set up at 100 μM for 48 h at 37 °C under shaking conditions. Data are presented as mean values +/− SD with all individual values plotted. **b** Quantification of ClearTau isoforms’ fibrillar widths. Two-way ANOVA with Tukey’s multiple comparisons test. Significant values are denoted by: ns *P* > 0.05, *P ≤ 0.05, **P ≤ 0.01, ***P ≤ 0.001, ****P ≤ 0.0001. Exact p-values can be found in the Source Data file. **c** Tau isoform incorporation into the pellet fraction. Data are presented as mean values +/− SD with all individual values plotted for three independent reactions. **d** Transmission electron microscopy assessment of ClearTau fibrils composed of different Tau isoforms. Close-ups of the boxed areas are in black squares. The experiment was repeated of minimum three independent times with consistent results. **e** CD spectra of the endpoint of the ClearTau fibrillization of all six Tau isoforms reveal the adoption of higher-order molecular structures compared to the starting isoforms’ monomers. **f** High magnification of the different isoform fibril morphologies. The experiment was repeated of minimum three independent times with consistent results. **g** SDS-PAGE gel visualization of Tau protein retainment in the supernatant (S) and incorporation into insoluble pellet (P). The experiment was repeated at a minimum of three independent times with consistent results. Source data are provided as a Source Data file.

Therefore, we first evaluated the applicability of our ClearTau method to the fibrillization of Tau 4R2N, 4R1N, 4R0N, 3R2N, 3R1N, and 3R0N proteins. Three independent ClearTau reactions were conducted for each of the six Tau isoforms, and resulting aggregates were evaluated using ThS assay, CD, and EM. Each Tau isoform was fibrillized as described previously (See Methods). The extent of fibrillization was assessed on the whole sample aliquots by ThS fluorescence assay before ultracentrifugation separating monomers and pellets (Fig. 4a), showing the signal for all Tau isoforms and repeats. However, we observed inter-isoform variability with 3R2N showing the highest ThS fluorescence. Then the fibrils were separated by ultracentrifugation to yield pure fibril fraction for further analyses. The quantification of the ClearTau fibril widths, at the widest part with respect to the twists (if present) of all Tau isoforms, showed the shortest isoform 3R0N having the narrowest fibrils at 12.49 ± 2.59 nm, followed by 3R1N at 14.49 ± 3.19 nm, 3R2N at 14.73 ± 2.55 nm, 4R2N at 15.20 ± 2.24 nm, 4R0N at 15.70 ± 3.04 nm, with the widest fibrils shown by the 4R1N isoform at 15.83 ± 3.49 nm (Fig. 4b, Supplementary Table 1).

The extent of monomer incorporation into fibrils showed high efficiency for 4R0N and all 3 R isoforms, as evident by the shift of Tau from soluble (S) to insoluble pellet (P) fractions isoforms (Fig. 4c, g Supplementary Fig. 5). Examination of the fibrils by EM revealed morphological differences between the fibrils derived from the various Tau isoforms (Fig. 4d, f). The 4R2N, 4R1N, and 4R0N samples formed long, flexible curved fibrils. 3R2N formed straight, more rigid fibrils. 3R1N and 3R0N showed curved, twisted fibrils. The 3R2N stood out with non-twisted straight fibrils, whereas all other isoforms’ twists differed in their periodicity and higher-order coiling, resulting in fibrils of differing conformations (Fig. 4f). The CD spectra of the aggregated solutions of the isoforms 4R2N and 3R1N demonstrated a signal loss, indicating the formation of structured insoluble material, whereas isoforms 4R1N, 4R0N, 3R2N, and 3R0N demonstrated the shift towards the higher-order secondary structure-containing species (Fig. 4e). Interestingly, the 3R2N showed the most prominent shift of the minimum towards 210 nm.

### Cryoelectron microscopy of ClearTau fibril structures

**Fig. 5:**
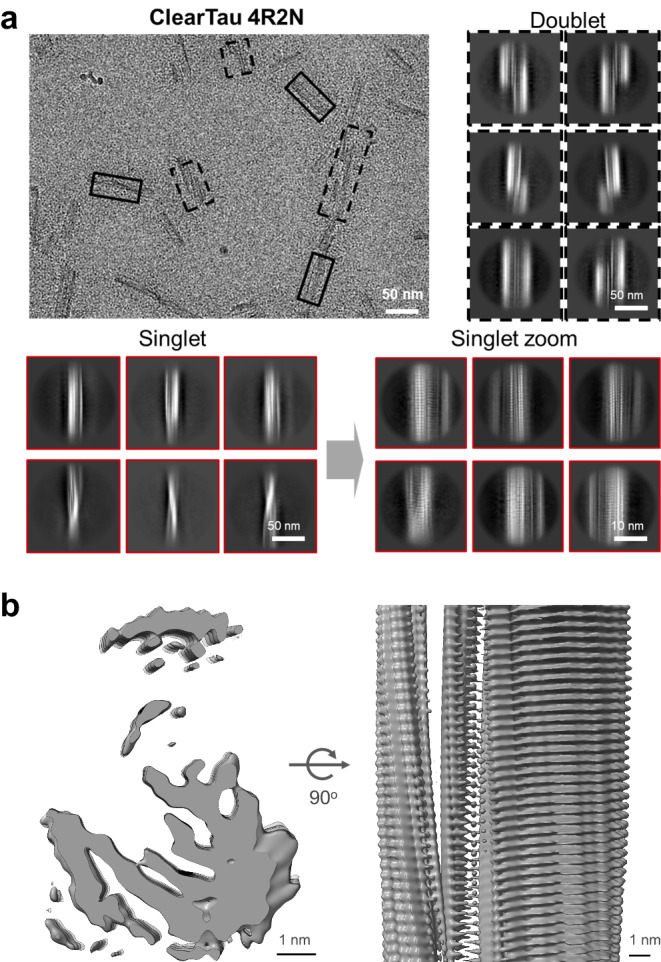
Cryo-EM micrograph of fibrils from ClearTau 4R2N. **a** Selected singlets and doublets are outlined with solid or dashed boxes respectively. Representative 2D class averages of singlets and doublets from large 900 pixel segments downscaled to 300 pixel with visible twist of the amyloid core. Representative 2D class averages of singlets from 300 pixel non-scaled segments with clear amyloid core and 4.77 Å separation of beta-strands. **b** Top sliced view and side view of the 3D reconstruction of ClearTau 4R2N singlet. The ClearTau 4R2N 3D reconstruction exhibits amyloid core stacking. Scale bar = 1 nm.

Next, we used cryo-EM to gain more insight into the ultrastructural properties of ClearTau fibrils composed of 4R2N and 3R2N Tau isoforms. Raw cryo-EM micrographs demonstrated that fibrils from ClearTau 4R2N were fragmented, featuring singlets and doublets with crossing-over of the composing filaments (Fig. 5a, Supplementary Fig. 5a–c). 3R2N fibrils were straight and fragmented, featuring the presence of singlets and doublets (Supplementary Fig. 5d), however, due to lack of helical symmetry the computational reconstruction of the fibrillar core has not been straightforward. Furthermore, an inspection of raw micrographs and 2D class averages revealed the presence of two polymorphs with different widths. The singlet polymorphs comprised long and 160 Å wide filaments with major and minor grooves and visible crossover representing 64 and 73% in all of the extracted segments for samples 3R2N and 4R2N, respectively. Visible crossover of 4R2N singlet fibrils allowed us to measure the corresponding helical twist of -0.928 degrees. The doublet polymorphs (36% for 3R2N and 27% for 4R2N) were short and 380 Å-wide filaments that appeared to be composed of two copies of 180 Å wide polymorphs zipped together with minor grooves. The data allowed a preliminary reconstruction of the 3D structure of the ClearTau 4R2N singlet fibrils at a moderate resolution (Fig. 5b). The results show that ClearTau 4R2N fibrils comprise amyloid core stacking, however the main chain was not resolved and the resolution must be improved.

These observations suggest that the absence of heparin at the protofibril interface allowed for the formation of fibrils composed of two intertwined symmetrical filaments - the doublets. Interestingly, the edges of the protofilaments exhibited different lengths and lacked a uniform flat edge (Supplementary Fig. 5d). It is possible that the formation of doublets is preceded by the formation of singlet fibrils, which then come together, and, depending on isoforms, either twist around each other or remain flat. These results demonstrate that our ClearTau method is highly efficient for all Tau isoforms and is conducive to forming symmetrical double-filament Tau fibrils. These findings are also in contrast to double-filament RNA-induced Tau polymorph, where the filaments formed asymmetrical structures, and included intercalation of RNA into the fibrils^27^. Symmetrical doublet fibrils thus far have not been demonstrated for conventional in vitro FFH-fibrillized 4R2N isoform using cryo-EM^24^, whereas 3R2N doublets detected previously^24^ appeared asymmetrical.

### ClearTau fibrillization of Tau isoform mixtures

**Fig. 6:**
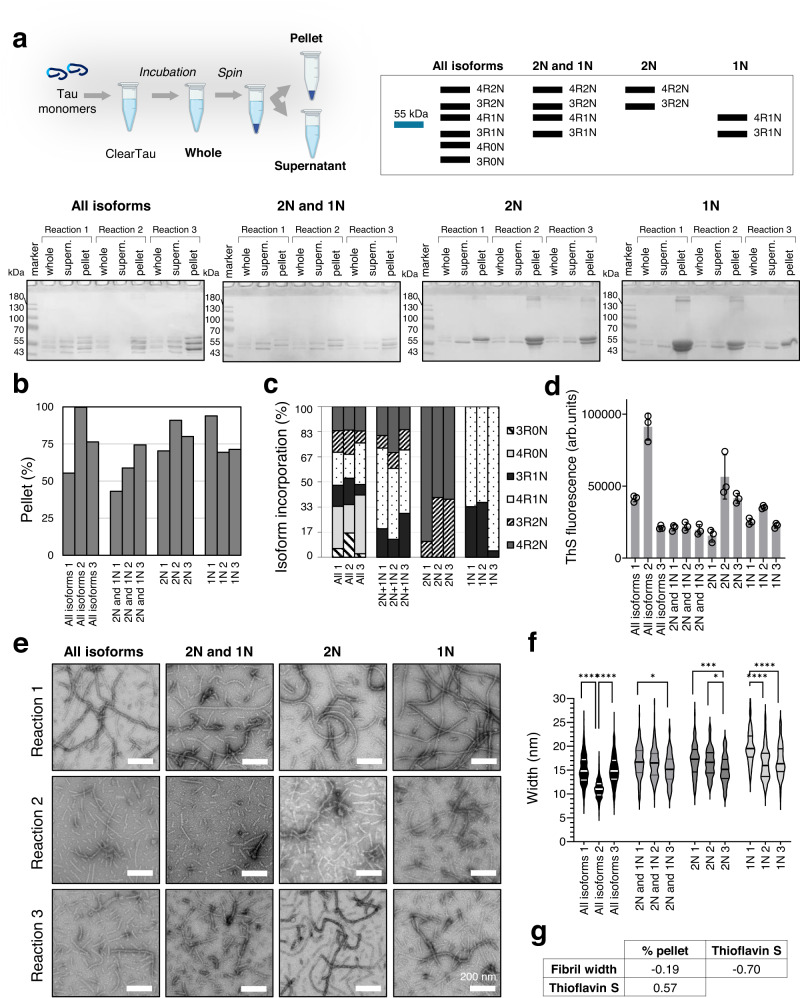
Characterization of ClearTau fibrils generated from Tau isoform mixtures. **a** Three independent ClearTau reactions (*n* = 3 biologically independent samples examined over 3 independent experiments) were set up to include equal amounts of indicated Tau isoforms for 48 h for each condition with three technical replicates each. The fibrils (pellet fraction) were isolated from the remaining monomers (supernatant fraction) by ultracentrifugation. On the right: expected banding patterns of SDS-PAGE gel Tau isoform under four reaction conditions. (See Supplementary Fig. 6). Created with BioRender.com. **b** Quantification of the percentage of the monomer incorporation into the fibrils for all reactions shows high efficiency as early as 48 h of the aggregation. **c** Representation of the quantification of the relative isoform incorporation into the fibrils for all repeats of the four mixtures. **d** ThS fluorescence of the endpoint fibrils for all reactions (*n* = 3 biologically independent samples examined over 3 independent experiments). Data are presented as mean values +/− SD with all individual values plotted. **e** Electron microscopy assessment of ClearTau fibrils prepared from the mixtures of Tau isoforms. All samples contain well-defined doublet fibrils, except the All isoforms (Sample 2), which appear to comprise straight, rigid singlet filaments. Scale bars = 200 nm. The experiment was repeated of minimum three independent times with consistent results. **f** Quantifications of the fibril widths for each repeat of the four mixtures. Two-way ANOVA with Tukey’s multiple comparisons test. Significant values are denoted by: ns *P* > 0.05, *P ≤ 0.05, **P ≤ 0.01, ***P ≤ 0.001, ****P ≤ 0.0001. Exact *p*-values can be found in the Source Data file. **g** Correlation analysis of fibril with ThS, monomer incorporation into the pellet, and ThS fluorescence. Source data are provided as a Source Data file.

The isoform composition of patient-derived pathological Tau fibrils is non-homogeneous and contains all six Tau isoforms, with ratios differing between Tauopathies^33^. Therefore, to determine if our method is suitable for modeling the complexity of Tau aggregation in the brain, we assessed the aggregation of four mixtures of Tau isoforms in varying compositions in the ClearTau method under the same conditions described above. The mixtures comprised equimolar amounts of all six Tau isoforms (All isoforms; 4R2N, 4R1N, 4R0N, 3R2N, 3R1N, 3R0N), isoforms containing 2 N and 1N repeats (2 N + 1 N; 4R2N, 4R1N, 3R2N, 3R1N), isoforms containing 2N repeats (2N; 4 R2N, 3 R2N), and isoforms containing 1N repeats (1N; 4R1N, 3R1N) in three individual reactions, (Reaction 1, 2 or 3) (Fig. 6a). All samples showed efficient fibrillization, where Tau was enriched in the pellet fractions (Fig. 6b). Interestingly, the relative incorporation of 4R-containing isoforms into the fibril-containing pellet fractions was consistently higher than 3R-containing isoforms (Fig. 6c, Supplementary Fig. 6). Notably, All isoforms (Reaction 2) showed complete incorporation of all six Tau isoforms into the fibrils.

Next, we assessed the binding of the fibrils generated in the different mixtures to ThS (Fig. 6d). All samples showed a ThS signal indicating a formation of the amyloid motif-containing fibrils. Notably, the All isoforms (Reaction 2) demonstrated the highest ThS signal, indicating potential morphological or ultrastructural differences from all other isoform mixtures’ fibrils. To further assess the morphology of the fibrils, EM imaging was performed (Fig. 6e). All isoforms (Reaction 1) contained flexible fibrils of 15.15 ± 3.03 nm in width, and Reaction 3 contained similarly bendy fibrils of 15.08 ± 3.00 nm in width. The All isoforms (Reaction 2), however, showed a predominantly homogeneous population of the thin, rigid fibrils 11.02 ± 1.83 nm in width, significantly narrower than the All isoforms (Reaction 1) and the All isoforms (Reaction 3) (Fig. 6f, Supplementary Table 2). 2 N + 1 N (Reactions 1, 2, and 3) contained thick, flexible fibrils; similar to the 2 N (Reactions 1, 2, and 3). 1 N (Reactions 1, 2, and 3), however, formed flat, right-hand twisting ribbon-like fibrils. The fibril width showed a strong negative correlation with the ThS signal, signifying those thinner, likely singlet fibrils have higher surface availability for the ThS dye binding (see Fig. 6g) and thus a high ThS binding/fluorescence.

Together, these data show that the ClearTau fibrilization method is suitable for investigating the aggregation of Tau isoform mixtures and allows for the efficient production of Tau fibril preparations composed of mixtures of Tau isoforms, providing further opportunities to investigate variable ratios of non-modified and modified Tau isoforms that more closely represent isoform profiles in the human pathologies.

### ClearTau fibrillization in the presence of co-factor molecules

Previous polyanion-based Tau aggregation methods did not allow for investigation into the effects of co-factors or other Tau ligands at different stages of Tau oligomerization and fibril formation because of competition with the polyanions or drugs in solution or their tight binding to Tau aggregates^26,34^. Our method addresses this limitation because the heparin is immobilized, and other co-factors or ligands could be added at different time points during the aggregation of Tau proteins without interference. Polynucleotide molecules, such as RNA^35^, and mononucleotide molecules, such as ATP^36^, contain a negative charge and have been used to induce aggregation of Tau in vitro. RNA has been detected associated with Tau-positive aggregates in human pathologies^37,38^. Furthermore, Tau-RNA interactions are important in liquid-liquid phase separation and the formation of condensates^39^. As shown previously, heparin could compete with the binding of RNA, displacing it from the growing Tau fibril^40^, likely due to partially-overlapping binding sites on Tau. Therefore, we investigated the interactions of these molecules, RNA and ATP, with Tau upon fibrillization, but in the absence of the free-floating polysaccharide heparin.

**Fig. 7:**
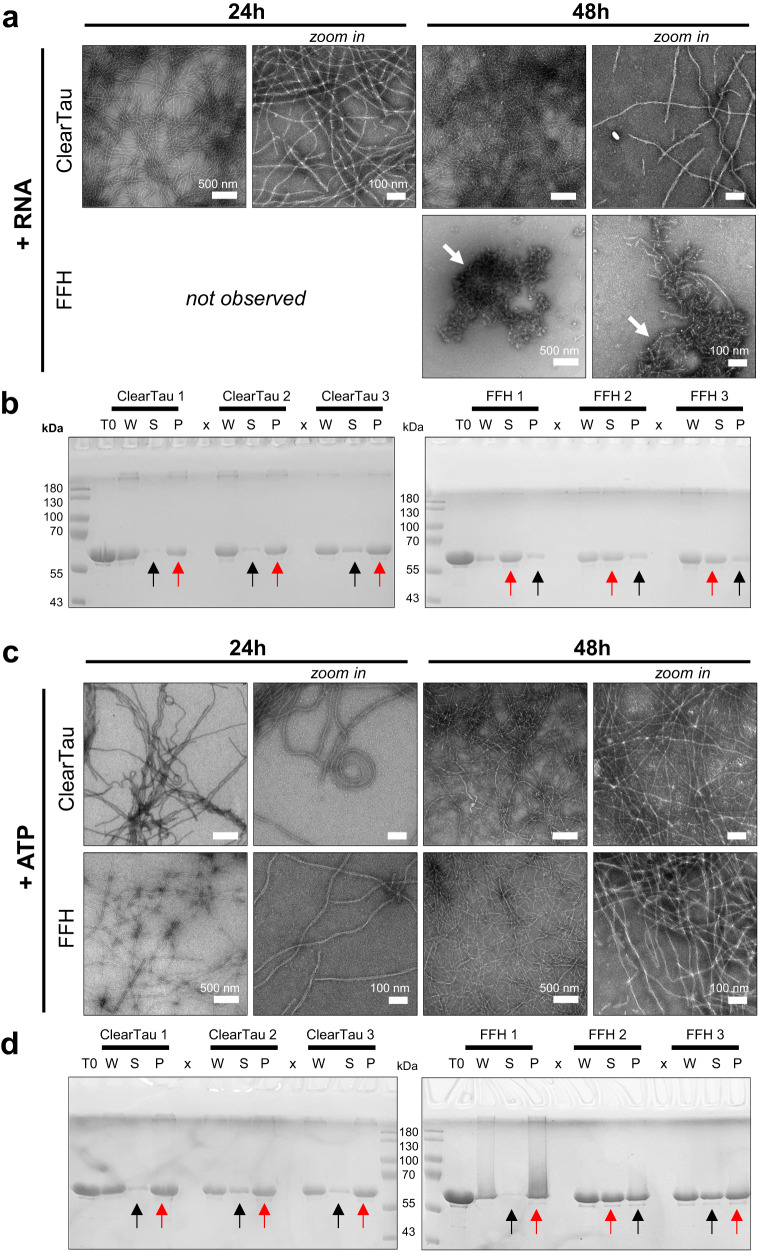
ClearTau fibrillization in the presence of co-factors. **a** Electron microscopy imaging and (**b**) SDS-PAGE gel of Tau fibrils in the presence of polyU RNA. **c** Electron microscopy imaging and (**d**) SDS-PAGE gel of Tau fibrils in the presence of adenosine triphosphate (ATP). T0 – time 0, W – whole sample, S – soluble fraction, P – pellet fraction. Both reactions were performed in triplicate independent aggregation reactions. Red arrows indicate thicker bands, and black arrows indicate narrower bands. Source data are provided as a Source Data file.

We assessed the aggregation of 4R2N Tau in the presence of other co-factors, polyuridylic acid (polyU, single-stranded RNA), or ATP by the ClearTau method or in the presence of FFH (1:4 ratio). The whole samples were imaged using EM midway through the reaction at 24 h and the endpoint at 48 h (Fig. 7a, c). Endpoint samples were fractionated to yield soluble supernatant (S) fractions containing monomeric Tau that was not incorporated into the fibrils, and pellet (P) fractions, containing fibrillized Tau. The samples were visualized using SDS-PAGE (Fig. 7b, d). In the presence of RNA, the EM imaging demonstrated the fibrillization of Tau by the ClearTau method, with numerous fibrils present at 24 h of the reaction (Fig. 7a). In contrast, FFH samples failed to aggregate at 24 h and showed indiscriminate amorphous aggregates and short fibrils at the later time point at 48 h (Fig. 7a, white arrows). In addition, the sedimentation assays showed efficient incorporation of Tau monomers into the pellet fraction (Fig. 7b, ClearTau, red arrows) in the presence of RNA, with a small amount of monomer present in the supernatant (Fig. 7b, ClearTau, black arrows) for ClearTau samples. In contrast, the FFH samples showed low levels of incorporation of Tau into the pellet fractions (Fig. 7b, FFH, red arrows), with most protein detected in the supernatant (Fig. 7b, FFH, black arrows). These observations illustrate the unique advantages of using our ClearTau method to investigate interaction between Tau and other ligands or aggregation co-factors and highlight the limitations of the standard FFH method. On the other hand, in the presence of mononucleotide ATP, both ClearTau and FFH samples showed fibrils both at 24 h and 48 h of aggregation reactions (Fig. 7c). The ClearTau fibrils appeared long, curly, flexible, and fuzzy. FFH samples showed long, more flat fibrils. Again, the sedimentation assays demonstrated high levels of Tau incorporation into the pellet in ClearTau samples (Fig. 7d, ClearTau, red arrows) with a small amount of Tau remaining in the supernatant (Fig. 7d, ClearTau, black arrows). However, the FFH samples showed high intersample variability, with the reaction FFH 1 demonstrating good incorporation of Tau into the pellet, whereas samples FFH 2 and FFH 3 showed lower incorporation of the protein into the fibril-containing fractions (Fig. 7d, FFH, red arrows). FFH 3 demonstrated a lower amount of protein in the pellet fraction (Fig. 7d, FFH, black arrows indicate lower, red arrows indicate the higher amount in the corresponding fraction).

These results demonstrate the higher reproducibility and efficient fibrillization of Tau in the presence of co-factors by the ClearTau method. EM imaging showed very long fibrils (upwards of 5 μm) bundled into flexible cable-like structures and a fuzzy appearance. Interestingly, biochemically the ClearTau samples form more insoluble aggregates upon both RNA and ATP addition and show a higher amount of fibrillar Tau compared to FFH reactions (Fig. 7b, d ClearTau). FFH samples demonstrated poor fibrillization in the presence of RNA and highly variable fibrillization in the presence of ATP. These results suggest potential interference of FFH with the co-factor or ligand molecules and their interactions impacting the Tau fibrillization propensity, as opposed to the ClearTau method.

### ClearTau 4R2N P301L fibrils are twisted and bind RNA

**Fig. 8:**
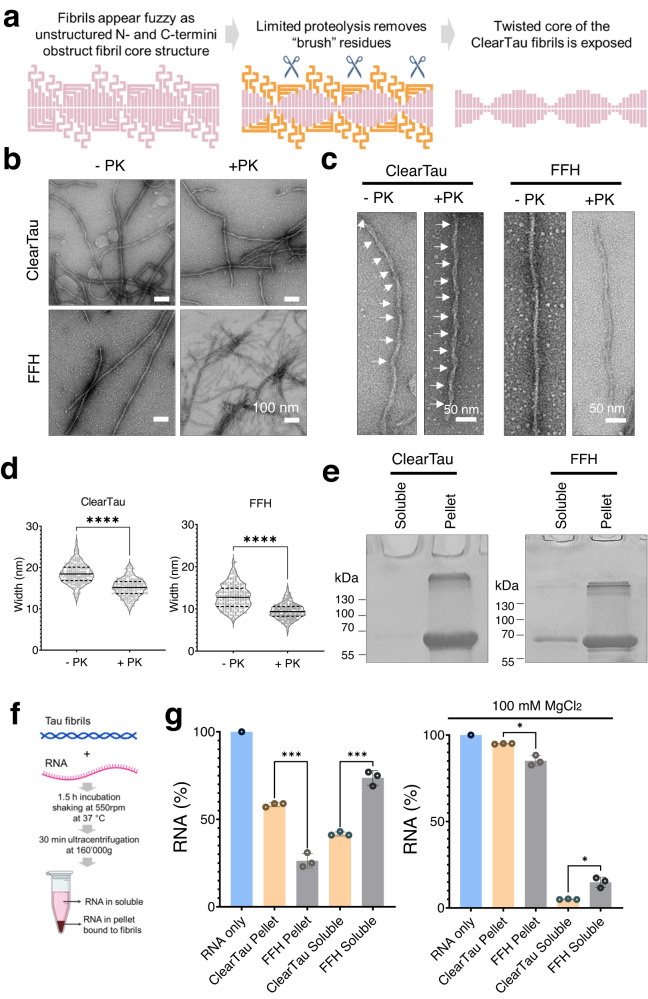
The ClearTau method application for fibrillization of full-length Tau 4R2N with mutation P301L. **a** Schematic of proteinase K limited proteolysis to remove the “brush” residues to reveal the fibril core structure. **b** EM of ClearTau and FFH-fibrillized Tau 4R2N P301L. Scale bars = 100 nm. The experiment was repeated of minimum three independent times with consistent results. **c** Proteolytic digestion of the fibrils by protease K (PK). Arrows point out the twists on the ClearTau fibrils, absent in the FFH samples. Scale bars = 50 nm. The experiment was repeated of minimum three independent times with consistent results. **d** Quantification of the widths of fibrils pre- and post-PK digestion, two-way ANOVA, significant values are denoted by: ns *P* > 0.05, *P ≤ 0.05, **P ≤ 0.01, ***P ≤ 0.001, ****P ≤ 0.0001. Exact *p*-values can be found in the Source Data file. **e** Representative gels demonstrating the monomer incorporation at 24 h aggregation reaction for Tau 4R2N P301L prepared by ClearTau or FFH methods. For complete gels and individual replicates see Supplementary Fig. 5a. The experiment was repeated of minimum three independent times with consistent results. **f** Schematic of RNA-binding assay workflow. **g** PolyU RNA-binding capacity of the 4R2N P301L fibrils prepared using ClearTau or FFH methods was assessed in the aggregation buffer, or in the higher ionic strength aggregation buffer (+100 mM MgCl_2_). *N* = 3 biologically independent samples examined over 3 independent experiments. Data are presented as mean values +/− SD with all individual values plotted. Two-way ANOVA with Tukey’s multiple comparisons test. Significant values are denoted by: ns *P* > 0.05, *P ≤ 0.05, **P ≤ 0.01, ***P ≤ 0.001, ****P ≤ 0.0001. Exact p-values can be found in the Source Data file. Source data are provided as a Source Data file.

Next, we extended our in vitro ClearTau method to aggregation of pathologically-relevant 4R2N Tau containing mutation P301L. P301L is associated with familial frontotemporal dementia linked to chromosome 17^41^, and P301L Tau readily forms fibrils in vitro in the presence of heparin, and our ClearTau method (see Supplementary Fig. 2). Therefore, we investigated its fibrillization and biochemical, RNA-binding, and ultrastructural properties in ClearTau and FFH systems. From the EM imaging, we noted that the ClearTau P301L fibrils indistinctly showed periodical twisting, whereas the FFH P301L samples showed flatter fibrils (Fig. 8). This led us to hypothesize that the Tau N- and C-termini that remain disordered may obstruct the visualization of the ultrastructure of the ordered amyloid core of the fibrils. Thus, we have employed the limited proteolysis approach (Fig. 8a) using 10 μM proteinase K digestion of the fibrils for 1 min to cleave off the disordered “brush” residues. After digestion, the ClearTau fibrils demonstrated the sharp outline of the periodically-twisted fibrillar core (Fig. 8b, c +PK), whereas the FFH fibrils were straighter. Further, the quantifications of the fibril widths pre- and post-proteolysis showed ClearTau fibrils consistently wider than FFH fibrils, suggesting differing fibrillar core morphologies (Fig. 8d and Supplementary Table 3). Biochemical fractionation by ultracentrifugation of the fibrils into soluble and pellet fractions revealed a consistently higher amount of monomer incorporation into the fibrils for the ClearTau samples than for FFH samples (Fig. 8e and Supplementary Fig. 7a).

Finally, we assessed the RNA-binding properties of the fibrils prepared by both methods by incubating fibrils with RNA and assessing the presence of RNA in the soluble and fibril-bound fractions. The results showed significantly higher binding of the polyU RNA to the ClearTau fibrils in both buffer conditions, with the higher ionic buffer strength resulting in a substantial increase in RNA binding to the fibrils (Fig. 8g). PolyA RNA binding was not significantly different between the ClearTau and FFH samples, with low binding levels in the aggregation buffer and high binding in the presence of 100 mM MgCl_2_. The yeast transfer RNA failed to bind either type of fibrils in the aggregation buffer or the presence of 100 mM MgCl_2_ (Supplementary Fig. 7b). These results further strengthen the versatility of the ClearTau method for use with different Tau proteoforms and in combination with downstream investigational methods.

### The ClearTau method enables the identification of small-molecule stabilizers of Tau oligomers and modulators of Tau fibrillization

**Fig. 9:**
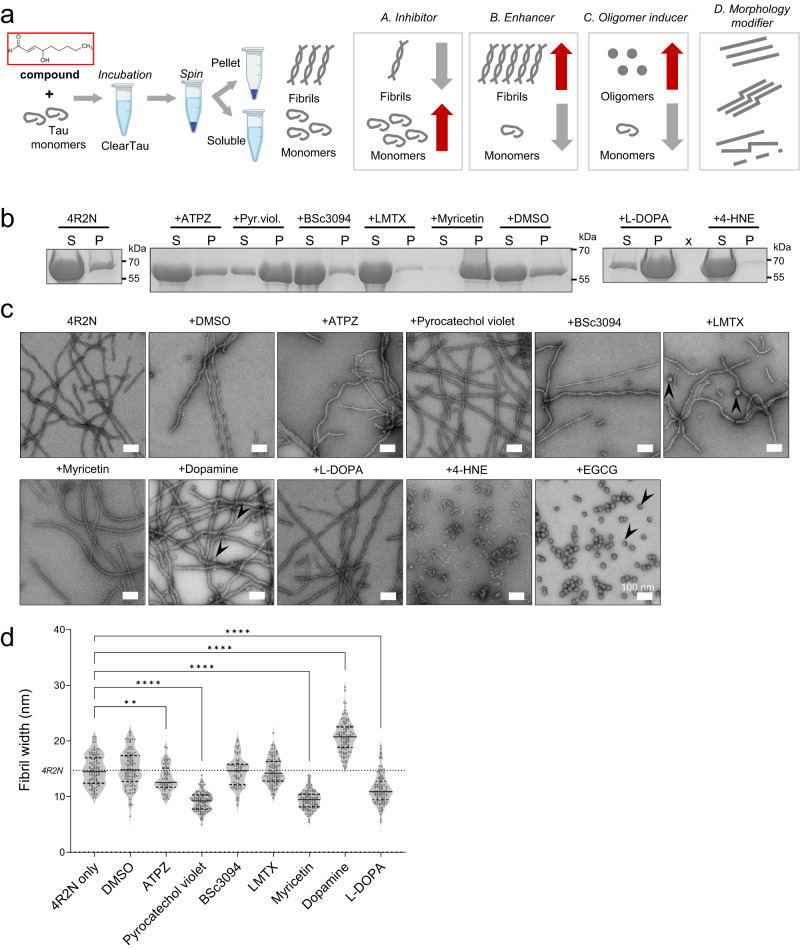
ClearTau method implementation for small molecule screening of the aggregation-modifying properties. **a** Schematic illustration of ClearTau method workflow implementation for screening of Tau aggregation inhibitors, enhancers, or oligomerization-inducing compounds and drugs. Created with BioRender.com. **b** SDS-PAGE illustrating the distribution of the Tau in the soluble (S) and pellet (P) fractions after incubation in the presence of small molecules. The experiment was repeated of minimum three independent times with consistent results. **c** Electron microscopy assessment of ClearTau fibrils and oligomers (arrowheads) formed in the presence of the compounds. The experiment was repeated at a minimum of three independent times with consistent results. **d** Quantifications of the fibril widths for each repeat of the four mixtures. Two-way ANOVA with Tukey’s multiple comparisons test. Significant values are denoted by: ns *P* > 0.05, *P ≤ 0.05, **P ≤ 0.01, ***P ≤ 0.001, ****P ≤ 0.0001. Exact *p*-values can be found in the Source Data file. Source data are provided as a Source Data file.

Next, we sought to determine if the ClearTau method could be used to screen and identify inhibitors of Tau oligomerization and fibril formation (Fig. 9a). Given that we can induce the misfolding and oligomerization of co-factor-free monomers and oligomers, we hypothesized that this assay could provide unique opportunities to identify compounds or drug-like molecules that can either stabilize Tau monomers or oligomers and inhibit their fibrillization. Such compounds could serve as powerful tools to test the role of Tau oligomerization and fibrillization in the pathogenesis of Tauopathies. As a proof of concept, we tested a panel of known modulators and/or inhibitors of Tau aggregation (Fig. 9b–d; ATPZ, pyrocatechol violet, BSc3094, LMTX, myricetin, L-DOPA, 4-HNE, and DMSO), in addition to other compounds that have been shown to inhibit the aggregation of other amyloid proteins.

The aldehyde 4-HNE and catechin EGCG compounds emerged as the most effective at preventing Tau fibril formation (Fig. 9b) and induced the formation of predominantly oligomeric Tau species as detected by EM (Fig. 9c). These results suggest that 4-HNE and EGCG inhibit Tau fibrillization by stabilizing oligomeric forms of the protein, consistent with their mode of action on other amyloid-forming proteins, including α-synuclein^42–47^. LMTX compound is a reduced form of methylene blue, a well-studied Tau aggregation inhibitor^48^. Although LMTX did not fully prevent fibril formation, most of the Tau protein remained in the Soluble fraction (Fig. 9b), indicating that it works by stabilizing soluble forms of the protein. Interestingly, the fibrils formed in the presence of LMTX were fragmented and had an aberrant morphology with regular bends or twisting, but only a few oligomers were present (Fig. 9c). The absence of visible oligomeric structures by TEM indicates that it either stabilized monomeric or low molecular weight oligomers of Tau. The compounds ATPZ, pyrocatechol violet, BSc3094, and myricetin did not prevent Tau fibril formation. In fact, all but BSc3094 increased the aggregation of Tau to varying levels, with myricetin being the most potent enhancer. All compounds had pronounced effects on the fibril morphologies, giving rise to fibrils with widths significantly smaller than observed for Tau fibrils formed in the absence of compounds (Fig. 9c, d, Supplementary Table 4). Morphologically, ATPZ and BSc3094 induced the formation of fibrils of varied morphologies and a high amount of fragmentation. On the other hand, pyrocatechol violet and myricetin induced the formation of uniform populations of thin fibrils, with wavy morphology in pyrocatechol violet, and sharply defined thin fibrils in myricetin reactions (Fig. 9c, d). As expected, fibrils formed in the presence of DMSO were similar to those formed by Tau alone. Interestingly, L-DOPA, a precursor to dopamine, was shown to enhance the shift of Tau into the Pellet fraction, with a low amount of protein remaining in the soluble state (Fig. 9b).

**Fig. 10:**
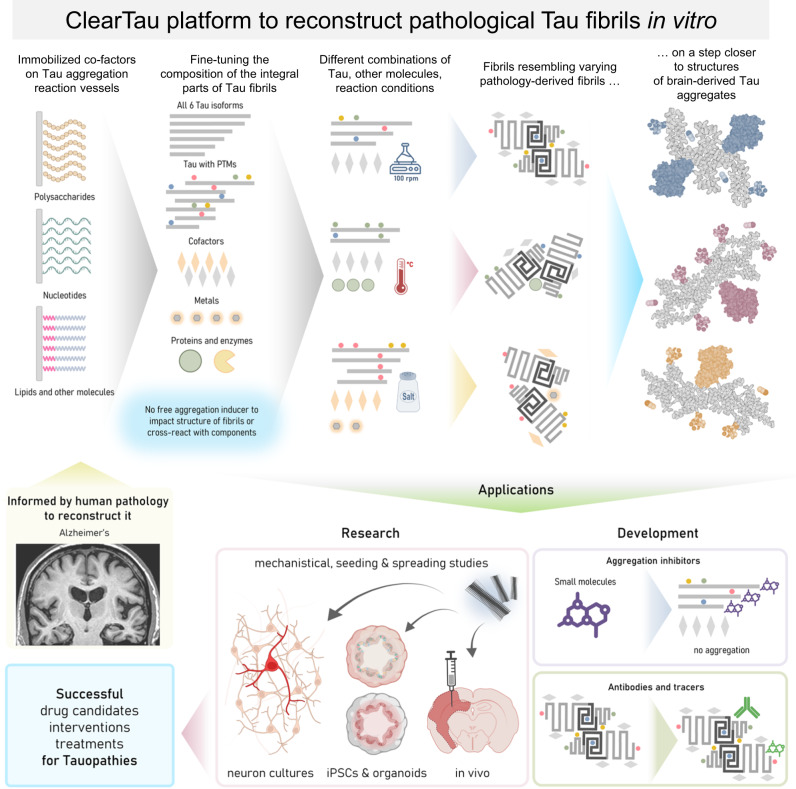
A schematic depiction of ClearTau platform to reconstruct pathology-resembling Tau fibrils in vitro for research into Tau aggregation processes and development of Tau fibril-targeting therapies and imaging agents. Informed by the insights about different types of co-factor molecules associated with Tau pathology in the brain or induce Tau pathology in vitro will guide the selection of different co-factors^79^ such as polysaccharides, nucleotides, lipids, and others that could be immobilized on the reaction vessel surface, allowing them to induce and/or catalyze Tau polymerization, but preventing them from integration into the growing Tau fibrils. The composition of Tau fibrillar aggregates can be tailored to contain specific Tau isoforms, PTMs, and other molecules, to more faithfully represent the pathological aggregates. The lack of free-floating co-factors prevents undesirable reactivity with the components in the aggregation reaction. Fine-tuning reaction conditions, such as temperature, agitation, or buffer, helps our ability to produce in vitro Tau fibrils more closely resembling the pathology-derived Tau aggregates. ClearTau methods can be applied to target the early stages of Tau aggregation for screening of aggregation-modifying compounds. ClearTau method and ClearTau co-factor-free aggregates can be used for research into Tau aggregation mechanisms, Tau seeding and spreading in in vitro and in vivo models, as well as to develop Tau fibril-targeting antibodies or PET tracer molecules. Created with BioRender.com.

The fibrils formed in the presence of L-DOPA were of uniform morphology, however, they were thinner, at 11.3 ± 2.72 nm, and visually straighter than control fibrils (14.7 ± 2.72 nm, Fig. 9c, d). In contrast to L-DOPA fibrils, the fibrils formed in the presence of dopamine showed wide cable-like bendy fibrils without a clear definition of the fibril surface. Some oligomeric species were also present. Here, we demonstrate that the Clear Tau methods can be used to screen and identify small molecule modifiers of the kinetics and morphology of Tau oligomerization and fibril formation. These findings suggest that it could also be used to screen putative natural co-factors to identify conditions that enable the conversion of wild-type of post-translationally modified forms of monomeric Tau into stable oligomeric forms or fibrillar structures that resemble those found in the brain of patients with Tauopathies (Fig. 10).

## Discussion

Attempts to develop methods for efficient production of clean co-factor-free fibrils from full-length Tau isoforms have thus far yielded unsatisfying results. One attempt to produce the co-factor-free Tau fibrils involved repeated cycles of Tau self-seeding reactions. However, the experimental timescales were limiting, for example, six cycles of self-repeated seeding at 5–7 days incubation per cycle equated to more than a month to produce the co-factor-free Tau fibrils^49^. Likewise, another recent attempt to produce the co-factor-free fibrils from a single Tau isoform using polytetrafluoroethylene beads suffered from long production timescales of  6 days^34^. Furthermore, the co-factor-free nature of the fibrils could not be ascertained due to the use of uncharacterized free-floating co-factor components in the aggregation buffer (protease inhibitor tablet containing aprotinin, bestatin, calpain I, calpain II, chymostatin, E-64, leupeptin, alpha-macroglobulin, pefabloc SC, pepstatin, TLCK-HCL, and trypsin inhibitors in undisclosed proportions) that could contribute to Tau fibrillization and intercalate into the growing fibrils^34^. In addition, a recent study attempted to generate Tau fibrils resembling patient-derived fibrils in vitro without the use of co-factors, but success in achieving this goal was limited to the truncated Tau constructs. Under all conditions tested, the unmodified full-length isoform 4R0N^50^ did not form fibrils. Therefore, efficient and cheap methods for producing co-factor-free, clean, unmodified full-length Tau fibrils are needed.

In this work, we present a ClearTau method for the production of large amounts of polysaccharide/co-factor-free Tau aggregates in vitro. We believe that ClearTau paves the way for more systematic approaches to investigate Tau aggregation mechanisms under conditions that preserve its interactome, thus enabling the production of Tau aggregates that recapitulate the biochemical and structural properties of brain-derived Tau aggregates. Covalent immobilization of polysaccharides, heparin, onto the surfaces of the vessels used to induce Tau aggregation results in the formation of high yields of pure, polysaccharide-free Tau protein fibrils. We demonstrate that fibrils produced using our method are morphologically-homomorphic within single isoforms and retain their structures upon association into the doublets; are amyloid reporter-dye positive; have high RNA-binding propensity; and are seeding-competent in Tau biosensor cells and human iPSC-derived neurons. Interestingly, in the absence of added cofactors, the ClearTau fibrils showed no seeding activity in vitro. These results highlight the critical role of the cellular environment as a key determinant of Tau seeding and pathology formation. They also suggest that interactions of Tau fibrils with specific combinations of cellular co-factors and/or PTMs of the fibrils may be necessary for Tau fibril seeding activity. Consistent with this hypothesis, recent studies on post-translationally modified α-synuclein fibrils showed that in vitro seeding aggregation studies do not predict α-synuclein fibril seeding activity in seeding-based cellular and in rodent models of Parkinson’s disease^51,52^. However, the in vitro ClearTau method provides a platform to screen for and identify PTMs or co-factors that regulate Tau seeding activity.

All six Tau isoforms, mixtures thereof, as well as truncated and mutant Tau, can be efficiently fibrillized using our method, including in the presence of other co-factor molecules, such as RNA and ATP. Further, we provided the proof-of-concept experiments illustrating the potential for extending the ClearTau method to screening applications aimed at identifying Tau aggregation inhibitors and modifiers. For example, we identified small molecules that inhibit or enhance Tau fibrillization, induce the formation of stable Tau oligomers, or produce fibrils with distinct morphologies, all in the absence of heparin or other co-factors. These compounds and conditions present unique opportunities to dissect the role of different aggregated forms of Tau and different Tau fibril strains in regulating its neurotoxicity, seeding, and spreading in the brain.

Our ClearTau aggregation method has multiple advantages over Tau aggregation methods containing free-floating polysaccharides as Tau aggregation co-factors. Lyophilized heparin has varying qualities from vendor to vendor, and batch-to-batch, such as a wide range of polysaccharide lengths, which impact the efficiency of Tau aggregation. Furthermore, the free-floating co-factors can tightly bind and integrate into Tau aggregation intermediates or the growing fibrils, thus impacting their structural features. The presence of polysaccharides in the mixture may have unfavorable interactions with the Tau-binding compounds. For example, Tau anti-aggregation compound leuco-methylthioninium molecule (TauRx Therapeutics)^26^ was found to bind to the heparin, therefore introducing artifacts into the experimental system, potentially resulting in significant economic and resource losses. In the cellular Tauopathy models, heparin has the potential to activate differential signaling pathways within cells that are not related to Tau fibril treatment^53–58^, and stimulate the aberrant modifications of Tau monomers^59^ or fibrils in cells. In animal models of Tau spreading, heparin can potentially interact with extracellular matrix constituents that influence Tau fibril spread and its cellular uptake^60,61^; elicit immune cell responses^62^ that are Tau-independent and impact the cardiovascular system cells and its components, resulting in polysaccharide-induced vasodilation^63^. Additionally, our results demonstrate that free-floating co-factor heparin can interact with other molecules, such as RNA, and impede Tau aggregation (Fig. 7).

In our ClearTau method, we solve these issues by removing heparin from the aggregation reaction to yield clean Tau fibrils at short time scales. The consistent Tau-to-immobilized polysaccharide ratio from one reaction to the next allows for optimizing the stoichiometry for best Tau aggregation at desired Tau concentrations. The high levels of interconversion of Tau monomer to fibril (up to 100%) are compatible with use with the high-cost, ultrapure, post-translationally modified, chemically modified, synthetic, or semisynthetic Tau proteins, in combination with other co-factors, and precious protein material only accessible at low amounts. Lack of the polysaccharide association with Tau protein fibrils allows for the downstream use in the ultrasensitive biochemical and biophysical assays, as well as in cell and in vivo studies. The ClearTau method utilizes minimal buffer conditions – up to containing PBS or dH_2_O only – thus enabling the identification and characterization of Tau interacting molecules without interference from other co-factors that may alter Tau fibril structures or interactome.

Recent advances in cryo-EM have provided remarkable insights into the structural diversity and landscape of Tau fibrils isolated from the brain of patients with different Tauopathies, thus enabling the shift towards a structure-based classification of these diseases^64^. Despite a wealth of information about the structure of Tau regions directly contributing to the core, the impact of the sequences in N- and C-termini, and mutations and PTMs in these regions on Tau fibrillization and final morphology of the fibrils remains largely unknown. These regions could also influence the binding of antibodies and other molecules, including PET tracers. Therefore, it is paramount to develop methods for generating fibrils composed of full-length Tau isoforms that resemble the morphological and structural properties of fibrils found in pathological Tau aggregates. Our preliminary cryo-EM results show that the fibrils generated using our ClearTau method exhibit structural features that are different from the known heparin-induced Tau fibrils. Further, ClearTau core structures appear reminiscent of pathology-derived Tau fibrils in an isoform-specific manner, but further studies are needed to determine the complete 3D structures of the pure monoisoform Clear Tau fibrils. Irrespective of whether the fibrils formed under the conditions used here are identical to those found in AD brain or not, we believe that the methods and data we present here pave the way for unprecedented opportunities to dissect the molecular and sequence determinants of Tau aggregation and pathology formation and bring us closer to reproducing the structural diversity of Tau fibrils isolated from different Tauopathies.

Accumulating evidence from mass spectrometry and cryo-EM studies of brain-derived Tau aggregates demonstrate the brain-derived Tau fibrils contain several post-translational modifications and non-proteinaceous compounds, which are thought to play a role in the initiation of Tau aggregation or influencing the final structure of the fibrils. Therefore, it could prove challenging to reproduce both the biochemical and structural diversity of brain-derived fibrils starting with unmodified Tau proteins and/or in the absence of specific cofactors. Previous methods used to induce Tau aggregation, precluded in vitro screening and identification of putative cofactors, because of interference by the polyanions used to induce Tau misfolding and aggregation. Our Clear Tau method makes such screening assays possible today. Furthermore, we and others have recently reported on the development of protein semisynthetic strategies that enable the generation of site-specifically modified forms of Tau bearing single or multiple PTMs in pure form^65–67^. We believe that combining these advances with insights into putative natural co-factors and PTMs that regulate Tau aggregation and pathology formation will provide a powerful platform (Fig. 10) to systematically evaluate and screen different combinations of Tau proteins and biochemical conditions and identify conditions that could enable reproducing the structure of pathological Tau aggregates from different Tauopathies (Fig. 10, Applications).

This will enable the generation of disease-relevant Tau targets to support drug discovery and to develop disease-specific Tau imaging agents. This is paramount to bridge the gap between the efficiency of Tau-targeting compounds in the laboratory, and the compounds’ effectiveness in patients suffering from AD and other Tauopathies. The ClearTau method is the stepping stone towards this goal.

## Methods

### Protein expression and purification

All proteins were expressed and purified as described previously^68^. Human 4R2N: Briefly, for the 4R2N isoform, the competent E. coli cells BL21 were transformed with plasmid pT7-7 SUMO-Tau human full-length 4R2N and incubated for 30 min on ice, heat-shocked for 45 s at 42 °C water bath, incubated on ice for 2 min. 300 µl of SOC outgrowth media (Thermo Fisher Scientific) were added, and the tube was incubated for 30 min @ 37 °C with shaking. Cells were plated on Luria-Bertani (LB) solid agar broth with kanamycin plates and incubated overnight in a 37 °C incubator for single colony growth. 20 ml of LB (Thermo Fisher Scientific) with 50 mg/L kanamycin were inoculated with a single bacterial colony and grown in the shaking incubator at 18 °C overnight. To 8 L of autoclaved and filtered LB antibiotic kanamycin was added to the final concentration of 50 mg/L, media was split into 4 × 2 L flasks, inoculated with an overnight starter culture of SUMO-Tau 4R2N transformed bacteria and grown at 37 °C at 4’000 g in the shaking incubator to the confluence of 0.6–0.9 density. The culture was induced for the protein production by adding isopropyl β- d-1-thiogalactopyranoside (IPTG, Thermo Fisher Scientific) to the final concentration of 0.4 mM and grown at 18 °C overnight in the shaking incubator. The culture was decanted into 1 L centrifuge tubes and centrifuged at 4’000 g for 30 min. 150 ml of lysis buffer (50 mM Tris pH7.5, 30 mM imidazole, 500 mM NaCl) with 1 mM phenylmethylsulfonyl fluoride (PMSF) and protease inhibitor cocktail (SigmaAldrich) were added, a bacterial pellet was solubilized fully, then sonicated on ice using a probe sonicator using the following protocol: 70% amplitude, 30 s on, 30 s off for 5 min. Sonicated lysate was centrifuged for 30 min at 10 °C at 40’000 g. The supernatant was filtered and loaded on a HisTrap HP 5 ml column (GE Healthcare). The purification was then performed using a linear elution gradient of His Trap Buffer A (50 mM Tris pH7.5, 30 mM imidazole, 500 mM NaCl) 100% to His Trap Buffer B (50 mM Tris pH7.5, 500 mM imidazole, 500 mM NaCl) 100%. The fractions were analyzed by SDS-PAGE, pooled accordingly, and cleaved by ULP1 enzyme (Thermo Fisher Scientific) for 1 h at room temperature (RT). The pooled fractions were then loaded on a reverse-phase HPLC C4 column (PROTO 300 C4 10 µm, Higgins Analytical; buffer A: 0.1% TFA in water, buffer B: 0.1% TFA in acetonitrile), and the protein was eluted using a gradient from 20 to 40% buffer B over 90 min (15 ml/min). Fractions were analyzed by mass spectrometry and ultra-performance liquid chromatography for purity and pooled accordingly. Protein samples were flash-frozen in liquid nitrogen and placed into a vacuum lyophilizer for 48 h to produce lyophilized protein powder. All other Tau isoforms and variants were produced following a similar protocol.

### ClearTau method

The ClearTau method was carried out in accordance with the procedures described in patent application No. 22204800.1 (priority founding). Briefly, monomeric human full-length 4R2N Tau was diluted to 100 μM (or other specified concentration) in phosphate buffer (10 mM Phosphate, 50 mM NaF, 0.5 mM fresh DTT) and added to a heparin-coated reaction tube (ThermoFisherScientific). The reaction mixture was incubated for 24 h with orbital shaking at 100 g (Peqlab, Thriller) at 37 °C. The reaction was ultracentrifuged using Beckman-Coulter ultracentrifuge at 100’000 g for 1 h at 4 °C, the supernatant was removed and discarded, and the pellet was washed twice with dH_2_O. The pellet containing fibrils was resuspended in dH_2_O to 100 mM, aliquoted to single-use aliquots, and stored at −80 °C. Analogous measurement procedures were followed for other Tau isoforms and mixtures thereof. Tau 4R2N P301L was aggregated in H_2_O.

### ClearTau seed preparation

The ClearTau fibrils were diluted to 10 μM in dH_2_O and sonicated at 70 % amplitude for 50 s with 1 s ON 1 s OFF cycle in-tube using UP200St with VialTweeter (Hielscher, USA). Seeds were characterized by electron microscopy.

### ThS fluorescence measurement

The ClearTau fibrils were diluted to 2.5 μM in dH_2_O and sonicated at 70 % amplitude for 50 s with 1 s ON 1 s OFF cycle in-tube using UP200St with VialTweeter (Hielscher, USA). 2.5 μM full-length Tau 4R2N monomer was used as a control. To the 100 μl reaction, 100 μl of ThS (10 μM) were added, yielding final protein concentrations of 1.25 μM. Single-timepoint ThS fluorescence was measured using 96 well clear bottom plates (Corning) set up in FLUOstar Omega microplate reader (BMG LABTECH, Germany) with excitation at 445 nm and emission at 485 nm was recorded. Four independent measurements were conducted in triplicates using ClearTau fibrils from two independent aggregation preparations. Raw fluorescence values were standardized to the blank reaction samples containing ThS. The plot represents the average of four experiments in triplicates, and the bars show the standard deviation. Analogous measurement procedures were followed for other Tau isoforms and mixtures thereof, the details for each indicated in the legends.

### ThS ClearTau seeding aggregation assay

The ClearTau seeds were prepared from Tau fibrils produced using the ClearTau method as described above. Reactions were set up in the clear bottom 96 well plates (Corning) as follows: Tau 4R2N monomer was diluted in the phosphate aggregation buffer (10 mM Phosphate, 50 mM NaF, 0.5 mM fresh DTT) to 10 μM. The ClearTau seed or free heparin sodium salt (Applichem GmbH) was added at a final concentration of 2.5 μM. The monomers without seeds were incubated with the buffer. A fresh ThS solution was added at 10 μM and the plate was sealed with clear film. Reactions were conducted in the FLUOstar Omega microplate reader (BMG LABTECH, Germany). The reading was taken from time 0 (corresponding to the maximum 20 min after the seed addition) every 10 min (1 cycle) for 19 h with shaking at 60 g for 10 min followed by the idle 10 min at 37 °C. 4 independent experiments were performed in triplicates for each condition. The ClearTau seeding values were standardized to the values derived from a reaction containing only seeds, but no monomer, heparin seeding values were standardized to the reaction containing heparin only and no monomer, monomer only reaction values were standardized to the reaction containing buffer, and ThS only. The plot represents average values for 4 experiments, and the bars represent the standard deviation.

### Negative stain transmission electron microscopy (EM)

2 μl of protein in solution were deposited on the glow-discharged Formvar/carbon-coated 200-mesh copper grids (Electron Microscopy Sciences) for electron microscopy, incubated for 5 min, washed three times in dH_2_O, and stained using 2% uranyl formate solution. Images were acquired using a Tecnai Spirit BioTWIN transmission electron microscope operated at 80 kV and equipped with a LaB6 filament and a 4 K × 4 K FEI Eagle CCD camera.

### Width quantification of fibrils

The ClearTau and FFH fibrils widths were quantified using EM images from at least 3 independent fibril preparations and independent EM grid preparations. Fibril widths were measured using ImageJ^69^ Measurement Tool (ImageJ, RRID: SCR_003070).

### Monomer – Fibril fractionation

The ClearTau reaction was ultracentrifuged using Beckman-Coulter ultracentrifuge at 100’000 g for 1 h at 4 °C. The supernatant was removed, and the remaining pellet was washed in dH_2_O twice and then resuspended in dH_2_O.

### SDS-PAGE protein assay

Twenty-five µg of total protein per well was loaded on fixed polyacrylamide concentration of 15 % SDS-PAGE gels (Invitrogen, Thermo Fisher Scientific) and run in MES buffer (Invitrogen, Thermo Fisher Scientific). The total protein content was visualized using the Coomassie protein stain.

### Biosensor (BS) cellular flow cytometry assay

Cell line TauRD P301S FRET Biosensor (CRL-3275™) was acquired from ATCC® and maintained in DMEM medium with 0.5% L-Glutamine, 0.5% penicillin-streptomycin antibiotic cocktail and 10 % fetal bovine serum supplementation (Gibco, Thermo Fisher Scientific). BS cells were plated in poly-L-lysine treated 6 well plates at a density of 100’000 cells/well. Cells were allowed to grow and divide in an incubator at 37 °C. BS cultures were allowed to reach a confluency of 50–60%. The ClearTau sonicated fibril seeds were incubated with Lipofectamine2000 at a 1:2 ratio weight to volume in OptiMEM (Gibco, Thermo Fisher Scientific). The cultures were transduced with ClearTau sonicated fibril seeds at amounts of 0.892 μg, 0.446 μg, and 0.223 μg of fibrils per 200’000 cells. Cells were exposed to the fibrils for 4 h, cultures were washed twice in phosphate-buffered saline (PBS, Gibco, Thermo Fisher Scientific) to remove all residual seeds, and further incubated for 96 h in standard medium to allow to have two cell division cycles. Cultures were washed once in PBS and dissociated using 200 μl Trypsin-EDTA 0.05% for 5 min at 37 °C. 150 μl of DMEM medium was added to each well to neutralize Trypsin action, cells were gently dissociated into single cells by pipetting, and they were transferred into Eppendorf tubes and centrifuged at 1000 g at room temperature for 5 min. The supernatant was removed, and cells were re-suspended in the 900 μl of 2% paraformaldehyde (Thermo Fisher Scientific) and incubated for 10 min, then pelleted by centrifugation at 1000 g 4 °C for 5 min. The supernatant was removed, and the pellet was re-suspended in HBSS.

FRET Flow cytometry protocol was adjusted from^70^. FRET detection was performed using BD LSR Fortessa with excitation-emission laser filters: 405 – 465/30, and 488 – 530/30, with the FRET signal detectable at 405 – 530/30 couple. Parental HEK293T cells were used to define the cell population on the SC-A vs FCS-A bivariate plot (Supplementary Fig. 9). Doublet events were excluded on FSC-H vs. FSC-A bivariate plot. Voltages were adjusted to exclude any signal on CFP, YFP, or FRET filters. Double-positive CFP-YFP BS cell population was defined by the negative Lipofectamine-only TauRD P301S FRET Biosensor cell line control sample. Compensation was adjusted to remove any bleed-through of CFP and YFP signal to the FRET channel. For data analysis, cell populations were gated to exclude the debris events and doublets. Negative control (Lipofectamine-only) was used to define a double-positive cell population, and spill-over to the FRET channel was excluded on CFP-FRET and YFP-FRET bivariate plots (See Supplementary Fig. 9). For each sample, the percent of FRET-positive events and the Median of fluorescence intensity was recorded, and the product was plotted to represent Integrated FRET Density (IFD). Three independent experiments were performed for each condition with a minimum of 100’000 events per sample recorded. The plot represents average measurements; bars represent standard deviation.

### Confocal imaging

For confocal imaging, TauRD P301S FRET Biosensor cells were plated at a low density (>5’000/well) in the 24 well plates on the poly-l-lysine-coated (3438-100-01, R&D Systems) glass coverslips and allowed to attach overnight. Cells were incubated in the HBSS (Gibco) overnight, and then the medium was changed to normal, and cells were allowed to double. Media was aspirated, cells were washed in 1× PBS, 10 μM ClearTau preformed seeds were added in OptiMEM (Gibco) for 4 h, then the media was aspirated and replaced by the standard media. Cells were allowed to have two cell divisions, after which they were washed in 1× PBS, and were fixed using a 4% formaldehyde solution for 20 min. Cells were washed twice in 1× PBS, and coverslips were mounted onto microscopy slides using a Molwiol mounting medium (Sigma-Aldrich). Confocal images were acquired using Zeiss LSM 700 microscope.

### Seeding experiments in hiPSC-derived cortical neurons

The human induced pluripotent stem cell lines used in this study were derived from the line iPSC0028, which was obtained from Sigma. The following biallelic genetic modifications were introduced as described^32^: microtubule-associated protein Tau (MAPT) knockout (KO Tau) and triple mutant MAPT IVS10 + 14, IVS10 + 16, P301S (TM Tau). hiPSCs were differentiated using a guided protocol based on various published protocols^32^ with slight modifications. Briefly, cultures were cultured in E8 Flex (ThermoFisher) on Matrigel (Corning)-coated cell culture ware. For differentiation, cells were harvested as single cells using Accutase (ThermoFisher) and seeded at 500,000 cells per cm^2^ onto 77.5 μg/mL Matrigel-coated 6-well plates in E8 Flex medium supplemented with the 10 μM ROCK inhibitor Y-27632 (Millipore). Protocols for differentiation into cortical neurons have been previously described^32^. 14 days after plating TM Tau and KO Tau hiPCS-derived cortical neurons were exposed to different amounts of recombinant Tau (P301L 4R2N) aggregates generated in the absence (ClearTau method) or presence (FFH) of heparin in solution. Three weeks later, the neurons were fixed and stained to detect endogenous Tau aggregates as described below.

Immunocytochemistry: Three weeks after incubation with Tau fibrils, the hiPSC-derived cortical neurons were washed twice with PBS and fixed in ice-cold methanol for 15 mins at −20 °C, followed by rinsing in PBS and blocking in 5% fetal calf serum (FCS), 5% BSA and 5% goat serum (Sigma) for 1 h at RT and overnight incubation with primary antibodies at 4 °C. After rinsing, secondary Alexa fluor 488-conjugated goat anti-mouse (Cat# A11001, Thermo Scientific, 1:500 dilution) and Alexa fluor 647-conjugated rabbit anti-chicken antibodies (Cat# 703-605-155, Jackson Laboratory,1:500 dilution) were applied for 2 h at RT. The following primary antibodies were used: MC1 antibody (kindly provided by Peter Davies to AbbVie through a Material Transfer Agreement with Feinstein Institute; 2 μg/mL dilution) to detect the insoluble tau aggregates and anti-MAP2 antibody (chicken, Abcam, #5392; 1:5000 dilution) to counterstain the neurites, Nuclei were counterstained by DAPI. Immunoreactivity was imaged in the Operetta CLS High-Content Analysis System (Perkin Elmer) using the 40× water immersion objective (25 fields of view per well, *z*-stack with six images with 0.5 μm step). Image analysis was carried out on the maximum intensity projection images using the Harmony Software (Perkin Elmer, version 4.9). The neuronal nuclei were detected based on a threshold of >0.4 and an area of 40 μm2. MAP2 area was determined by a common threshold of 0.5 (as calculated by the Harmony software). The endogenous Tau aggregates were detected using the spot detection method B with a detection sensitivity of 0.2 and a split sensitivity of 0.5 (as calculated by the Harmony software). Thereafter, the selected Tau aggregates were further filtered by a relative, as well as corrected and uncorrected spot intensity. Based on these features, the MC1+ area (>2 μm^2^) was calculated and normalized to the number of neuronal nuclei.

Symmetric ELISA: hiPSC-derived neurons were cultured in 96-well plate format and seeded with recombinant P301L 4R2N Tau aggregates as described above. Cells were lysed in 50 μl/well Triton buffer 150 mM NaCl; 20 mM Tris, pH 7.5; 1 mM EDTA; 1 mM EGTA; 1% Triton-X-100; 1× cOmplete Protease inhibitors; 1× PhosStop Phosphatase inhibitors (Roche) and diluted 50–150-fold in assay buffer (20 mM NaH_2_PO_4_ pH 7.4, 140 mM NaCl, 0.05% Tween 20, 0.1% BSA) to fall within the experimentally validated linear range of the assay. ELISA plates (Maxi Sorp, Thermo Scientific) were coated overnight at 4 °C with 100 μl Tau-12 capture antibody per well (2 μg/ml; Biolegend, cat. no 806502) in 20 mM NaH_2_PO_4_ pH 7.4, 140 mM NaCl, 20% glycerol. Plates were rinsed with 250 μl/well wash buffer (20 mM NaH_2_PO_4_ pH 7.4, 140 mM NaCl, 0.05% Tween 20) and blocked for 1.5 h at RT with 250 μl/well-blocking buffer (20 mM NaH_2_PO_4_ pH 7.4, 140 mM NaCl, 0.05% Tween-20, 20% glycerol, 2% BSA). After rinsing, 100 μl/well samples were added and incubated for 2 h at RT. After rinsing, 0.1 mg/ml Biotin-Tau 12 (100 μl/well; BioLegend) was added for detection and incubated for 1 h at RT followed by Pierce Streptavidin poly-HRP conjugate was used (Thermo Scientific, diluted 1:10 000 in assay buffer) for 1 h at RT. TMB ELISA substrate (100 μl/well; KEM EN TEC Diagnostics) was incubated for 10 min in the dark for signal detection. The reaction was stopped by 100 μl/well 0.18 M H_2_SO_4_, and the absorbance was read at 450 nm on an Anthos LEDetect microplate reader (Anthos Mikrosysteme GmbH, Frisoythe, Germany).

### Circular dichroism (CD) spectroscopy

CD spectra were recorded on a Jasco J-815 CD spectrometer operated at 20 °C. To minimize buffer absorption, the samples were diluted with 1:50 deionized H_2_O. CD spectra were acquired from 190 nm to 260 nm at a scan rate of 50 nm/min and in increments of 0.2 nm. For each sample, three to four spectra were averaged and smoothed using binomial approximation.

### Cryoelectron microscopy

The fibrils from ClearTau 3R2N and 4R2N proteins were screened with negative staining (NS) TEM for fibril concentration and morphology. Aliquots of optimized fibril samples were applied onto gold Ultrafoil 1.2/1.3 grids, and plunge frozen in liquid ethane. Frozen cryo-EM grids were imaged on a ThermoScientific 200 kV Glacios on a K3 electron counting direct detection camera (Gatan Inc.) in counted (non-CDS) mode (50 fractions) using the SerialEM^71^ at a physical pixel size 1.113 Å for 3R2N fibrils and 0.878 Å for 4R2N fibrils, and a total dose of 50 electrons per square angstrom (e-/Å2) for each exposure. After inspection, best 6021 (3R2N) and 3331 (4R2N) aligned, CTF-estimated and dose-weighted movies were selected from FOCUS7^72^ for further processing. Several hundreds of representative non-overlapping filaments were manually selected using the e2helixboxer.py from EMAN2^73^. Dose-weighted averages were denoised with JANNI, and subjected for the semi-automated filaments tracing with crYOLOv1.7.5^74^. Filaments start-end coordinates in STAR format were imported into RELIONv3.1^75,76^, and 1,842,783 3R2N and 570,876 4R2N segments were extracted with box size of 300 pixels and subjected for reference-free 2D classification in cryoSPARCv3.2^77^. Several rounds of 2D classifications allowed to select only particles with clear 4.77 Å beta-strand separation along the fibril axis, measured from Fourier amplitudes of the 2D class average. To separate singlet and doublet fibrils, helical segments were re-extracted with larger box size of 900 pixels, re-scaled to 300 pixels and subjected for reference-free 2D classification in cryoSPARC. 2D class averages corresponding to singlets and doublets were separated and further classified. Visible crossover of 4R2N singlet fibrils allowed to measure the corresponding helical twist of −0.928°. Best segments corresponding to 4R2N singlets were re-extracted with box size of 600 pixels and subjected for helical reconstruction in cryoSPARC with 4.77 Å helical rise and −0.928° helical twist. Resulted helical 3D reconstruction exhibit amyloid core stacking; however the main chain is not resolved. The resolution improvement should be improved further.

### Co-factor aggregation

To aggregate 4R2N Tau in the presence of co-factor polyuridylic acid single-stranded RNA (P9528, Sigma-Aldrich), or adenosine triphosphate (987-65-5, SERVA/Cayman chemical) by ClearTau method or in the Eppendorfs in the presence of FFH, Tau 4R2N was added at 10 μg/ml to 1 mM of co-factor molecules. FFH was added at a 1:4 ratio. The reactions were set up in triplicates and incubated at 37 °C with orbital shaking at 100 g for 48 h. 100 μl of endpoint reactions (W) were ultracentrifuged using Beckman-Coulter ultracentrifuge at 100’000 g for 30 min at 4 °C. The supernatant (SN) was removed, remaining pellet (P) was washed in PBS twice, then resuspended in 100 μl PBS to yield a fraction containing fibrils. 10 μl of the fractions were mixed with 10 μl 2X Laemmli buffer, and 2 μl per well was loaded on the SDS-PAGE as described above.

### Proteinase K digestion of 4R2N P301L fibrils

10 μM of Tau 4R2N P301L was fibrillized in the ClearTau method and the presence of FFH for 24 h. 300 μl of samples were ultracentrifuged, the supernatant removed and the pellet resuspended in 300 ul of PBS. 50 μl of samples were digested by proteinase K (39450-01-6, Invitrogen) at 10 μg/ml for 1 min. The reactions were quenched by the PMSF at 0.3 mM. The samples were imaged using electron microscopy as described above.

### RNA-binding assay

Tau 4R2N P301L was fibrillized by the ClearTau method and the presence of FFH for 24 h. 300 μl of samples were ultracentrifuged, the supernatant removed, and the pellet resuspended in 300 μl of PBS. A total of 10 µg/mL of polyU RNA (P9528, Sigma-Aldrich), polyA RNA (P9403, Sigma-Aldrich), or yeast tRNA (R1753, Sigma-Aldrich) were added to 100 µL of 5 µM fibrils in the aggregation assay buffer (Phosphate buffer, pH 7.4). The fibril/RNA mixture was incubated at 37 °C with 55 g shaking for 1.5 h. After the incubation, the 80 μL of fibril/RNA-mixtures was centrifuged at 160’000 g at 37 °C for 30 min in an ultracentrifuge. The supernatant was removed, and the pellet was resuspended in 80 µL of aggregation assay buffer with 2% SDS. The concentration of RNA in the supernatant and the pellet (resuspended) was calculated using a Nanodrop One spectrophotometer (Thermo Fischer Scientific)^34^. Spectra were baseline corrected using the buffer as a reference. The concentrations of RNA were calculated from a sample of 10 µg/mL RNA in 100 µL of aggregation buffer. Measurements were performed with three independently prepared samples in each case.

### ClearTau method platform for screening of Tau aggregation inhibitors, enhancers, and structure modifiers

Monomeric human full-length 4R2N Tau was obtained as described previously and was diluted to 10 μM in phosphate buffer (10 mM Phosphate, 50 mM NaF, 0.5 mM fresh DTT) to obtain a suspension of the monomers in aqueous solution in an Eppendorf tube. 10 μM of the small molecules ATPZ (580222, Sigma), pyrocatechol violet (P7884, Sigma), BSc3094 (B7937, Sigma), LMTX (S7762, Selleckchem), myricetin (M6760, Sigma), DMSO (D841, Sigma), dopamine (H8502, Sigma), L-DOPA (D9628, Sigma), 4-HNE (32100, Cayman), EGCG (E4143, Sigma) were added to monomer, resulting in at 1:1 ratio. The reactions were then transferred to the ClearTau tubes at 200 μl and were placed on an orbital shaker at 100 g at 37 °C for 48 h aggregation. The reaction mixtures were then ultracentrifuged using Beckman-Coulter ultracentrifuge at 100’000 g for 1 h at 4 °C, the supernatant was removed and saved as a Soluble fraction, and the pellet was washed twice with dH_2_O. The pellet containing fibrils was resuspended in dH2O to 200 μl and labeled Pellet fraction. For EM, 5 μl of the Pellet was loaded as described below. For SDS-PAGE, 10 μl of samples and 10 μl of Laemmli buffer were added, and 5 or 10 μl of each sample was loaded on 15/5% PAA gel. Total protein was visualized by Coomassie stain. The quantification of the soluble and pellet fraction ratios was performed using ImageJ^69^ Gel Tool (ImageJ, RRID: SCR_003070).

### Statistical analyses and data visualizations

All statistical analyses and data visualizations were performed using GraphPad Prism 9 software (San Diego, USA) and Microsoft Office Suite tools (USA). Atomic visualizations were performed using PyMOL software^78^. Created with BioRender.com (Academic License, agreement number EB25FP2AOB), original graphics were made and assembled using MS Office tools.

### Reporting summary

Further information on research design is available in the Nature Portfolio Reporting Summary linked to this article.

### Supplementary information

Supplementary Information

Reporting Summary

### Source data

Source Data

## Data Availability

Source data are provided with this paper in the Source Data file. Source data are provided with this paper.
