# Author Correction: Fully co-factor-free ClearTau platform produces seeding-competent Tau fibrils for reconstructing pathological Tau aggregates

**DOI:** 10.1038/s41467-024-48976-w

**Published:** 2024-05-30

**Authors:** Galina Limorenko, Meltem Tatli, Rajasekhar Kolla, Sergey Nazarov, Marie-Theres Weil, David C. Schöndorf, Daniela Geist, Peter Reinhardt, Dagmar E. Ehrnhoefer, Henning Stahlberg, Laura Gasparini, Hilal A. Lashuel

**Affiliations:** 1 Laboratory of Molecular and Chemical Biology of Neurodegeneration, Institute of Bioengineering, School of Life Sciences, Ecole Polytechnique Fédérale de Lausanne, CH-1015 Lausanne, Switzerland; 2 Laboratory of Biological Electron Microscopy, Institute of Physics, School of Basic Sciences, Ecole Polytechnique Fédérale de Lausanne, CH-1015 Lausanne, Switzerland; 3 Biological Electron Microscopy Facility, School of Life Sciences, Ecole Polytechnique Fédérale de Lausanne, CH-1015 Lausanne, Switzerland; 4 Neuroscience Discovery, AbbVie Deutschland GmbH & Co KG, Knollstrasse, 67061 Ludwigshafen, Germany; 5 Department of Fund. Microbiology, Faculty of Biology and Medicine, University of Lausanne, CH-1015 Lausanne, Switzerland

**Keywords:** Assay systems, Protein aggregation

Correction to: *Nature Communications* 10.1038/s41467-023-39314-7, published online 4 July 2023

The original version of this Article contained an erroneous main Fig. 5, where cryo-EM 2D classes and the structure of alpha-synuclein fibrils were reproduced in panels a and b, respectively. The correct version of Fig. 5 replaces the previous incorrect version and reproduces the cryo-EM data for Clear 4R2N Tau, showing in panel b the structure that could be derived from this sample.

The caption of the original version of Fig. 5 incorrectly read: ‘**Cryoelectron microscopy image processing and 3D model generation for ClearTau 4R2N fibrils. a** 2D classes were performed with a 612 Å box size (blue) depicting 4R2N fibrils formed by two protofilaments crossing each other. Pink outlines small 2D classes, a 3D reference created by Inimodel2D, top view, and 2D cross-section images are shown. **b** 3D cross-sections and side view of the 3D reconstruction. The ClearTau 4R2N fibrils comprise two filaments, with a gap at approximately 0.7 nm width. Scale bar = 1 nm’.

The correct version of Fig. 5 caption replaces the incorrect version and reads: ‘**Cryo-EM micrograph of fibrils from ClearTau 4R2N. a** Selected singlets and doublets are outlined with solid or dashed boxes respectively. Representative 2D class averages of singlets and doublets from large 900 pixel segments downscaled to 300 pixel with visible twist of the amyloid core. Representative 2D class averages of singlets from 300 pixel non-scaled segments with clear amyloid core and 4.77 Å separation of beta-strands. **b** Top sliced view and side view of the 3D reconstruction of ClearTau 4R2N singlet. The ClearTau 4R2N 3D reconstruction exhibits amyloid core stacking. Scale bar = 1 nm.’

The original version of this Article also contained an error in the “**Cryoelectron microscopy of ClearTau fibril structures**” Results section, which incorrectly read ‘The singlet polymorphs comprised long and 160 Å wide filaments with major and minor grooves and visible crossover representing 64 and 73% in all of the extracted segments for samples 3R2N and 4R2N, respectively. The doublet polymorphs (36% for 3R2N and 27% for 4R2N) were short and 380 Å-wide filaments that appeared to be composed of two copies of 180 Å wide polymorphs zipped together with minor grooves. The data allowed a partial reconstruction of the 3D structure of the ClearTau 4R2N fibrils at a final resolution of 3.1 Å (Fig. 5b). The results show that ClearTau 4R2N fibrils comprise two filaments crossingover, with the gap between the filaments measuring approximately 0.7 nm in width. It was possible to reconstruct the 3D structure of the ClearTau 4R2N isoform fibrils at 3.1 Å ([https://www.ebi.ac.uk/pdbe/entry/emdb/EMD16812]), however, the resolution must be improved.’

The correct version adds ‘Visible crossover of 4R2N singlet fibrils allowed us to measure the corresponding helical twist of −0.928 degrees.’ after ‘respectively’. The correction version also replaces the previous incorrect sentence ‘The data allowed a partial reconstruction of the 3D structure of the ClearTau 4R2N fibrils at a final resolution of 3.1 Å (Fig. 5b). The results show that ClearTau 4R2N fibrils comprise two filaments crossingover, with the gap between the filaments measuring approximately 0.7 nm in width. It was possible to reconstruct the 3D structure of the ClearTau 4R2N isoform fibrils at 3.1 Å, however, the resolution must be improved.’ with ‘The data allowed a preliminary reconstruction of the 3D structure of the ClearTau 4R2N singlet fibrils at a moderate resolution (Fig. 5b). The results show that ClearTau 4R2N fibrils comprise amyloid core stacking, however the main chain was not resolved and the resolution must be improved’.

The original version of this Article also contained an error in the “**Cryoelectron microscopy**” paragraph of the Methods section, which incorrectly read ‘After inspection, the best 6021 (3R2N) and 3331 (4R2N) aligned, CTF-estimated, and dose-weighted movies were selected from FOCUS^72^ for further processing. Fibrils were selected manually from aligned micrographs, and 1,842,783 3R2N and 570,876 4R2N segments were extracted with a box size of 300 pixels and subjected to reference-free 2D classification in cryoSPARCv3.2^73^. Several rounds of 2D classifications allowed to select only particles with clear 4.77 Å beta-strand separation along the fibril axis, measured from Fourier amplitudes of the 2D class average. To separate singlet and doublet fibrils, helical segments were re-extracted with a larger box size of 900 pixels, rescaled to 360 pixels, and subjected for reference-free 2D classification in cryoSPARC and RELION. 2D class averages corresponding to singlets and doublets were separated and further classified. 4R2N data were further refined, both cylinder and initial model by IniModel2d converged to the same structure. After a couple of rounds of 3D classification and refinement, pseudo-21 screw symmetry was applied. Bayesian polishing and CTF refinement were applied, and the final reconstruction was post-processed with a soft-edge mask and a sharpening B-factor of −70 Å^2^. The resolution was estimated as 3.1 Å from the Fourier shell correlation (FSC) at 0.143. EMDB ID: EMD-16812.’.

The correct version replaces this sentence with ‘After inspection, best 6021 (3R2N) and 3331 (4R2N) aligned, CTF-estimated and dose-weighted movies were selected from FOCUS^72^ for further processing. Several hundreds of representative non-overlapping filaments were manually selected using the e2helixboxer.py from EMAN2^73^. Dose-weighted averages were denoised with JANNI, and subjected for the semi-automated filaments tracing with crYOLOv1.7.5^74^. Filaments start-end coordinates in STAR format were imported into RELIONv3.1^75,76^, and 1,842,783 3R2N and 570,876 4R2N segments were extracted with box size of 300 pixels and subjected for reference-free 2D classification in cryoSPARCv3.2^77^. Several rounds of 2D classifications allowed to select only particles with clear 4.77 Å beta-strand separation along the fibril axis, measured from Fourier amplitudes of the 2D class average. To separate singlet and doublet fibrils, helical segments were re-extracted with larger box size of 900 pixels, re-scaled to 300 pixels and subjected for reference-free 2D classification in cryoSPARC. 2D class averages corresponding to singlets and doublets were separated and further classified. Visible crossover of 4R2N singlet fibrils allowed to measure the corresponding helical twist of −0.928°. Best segments corresponding to 4R2N singlets were re-extracted with box size of 600 pixels and subjected for helical reconstruction in cryoSPARC with 4.77 Å helical rise and −0.928° helical twist. Resulted helical 3D reconstruction exhibit amyloid core stacking; however the main chain is not resolved. The resolution improvement should be improved further.’

The original version of this Article omitted four references to previous works in:

73. Tang, G. et al. EMAN2: an extensible image processing suite for electron microscopy. *J. Struct. Biol*. **157**, 38–46 (2007).

74. Wagner, T. et al. SPHIRE-crYOLO is a fast and accurate fully automated particle picker for cryo-EM. *Commun. Biol*. **2**, 1–13 (2019).

75. Scheres, S. H. W. RELION: implementation of a Bayesian approach to Cryo-EM structure determination. *J. Struct. Biol*. **180**, 519–530 (2012).

76. Zivanov, J. et al. New tools for automated high-resolution cryo-EM structure determination in RELION-3. *eLife* **7**, e42166 (2018).

These have been added as references 73, 74, 75, and 76 at the “Cryoelectron microscopy” paragraph of the Methods section: ‘Several hundreds of representative non-overlapping filaments were manually selected using the e2helixboxer.py from EMAN2^73^. Dose-weighted averages were denoised with JANNI, and subjected for the semi-automated filaments tracing with crYOLOv1.7.5^74^. Filaments start-end coordinates in STAR format were imported into RELIONv3.1^75,76^, and 1,842,783 3R2N and 570,876 4R2N segments were extracted with box size of 300 pixels and subjected for reference-free 2D classification in cryoSPARCv3.2^77^.’

The original version of this Article contained an error in the data availability statement, which reports the EMDB accession code for ClearTau 4R2N CryoEM structure as ‘EMD ID:16812’, this has now been removed due to the error in the structure.

These errors have been corrected in both the PDF and HTML versions of the Article.

The original and corrected versions of Fig. 5 are reproduced within this correction.

The original version of Fig. 5 is:
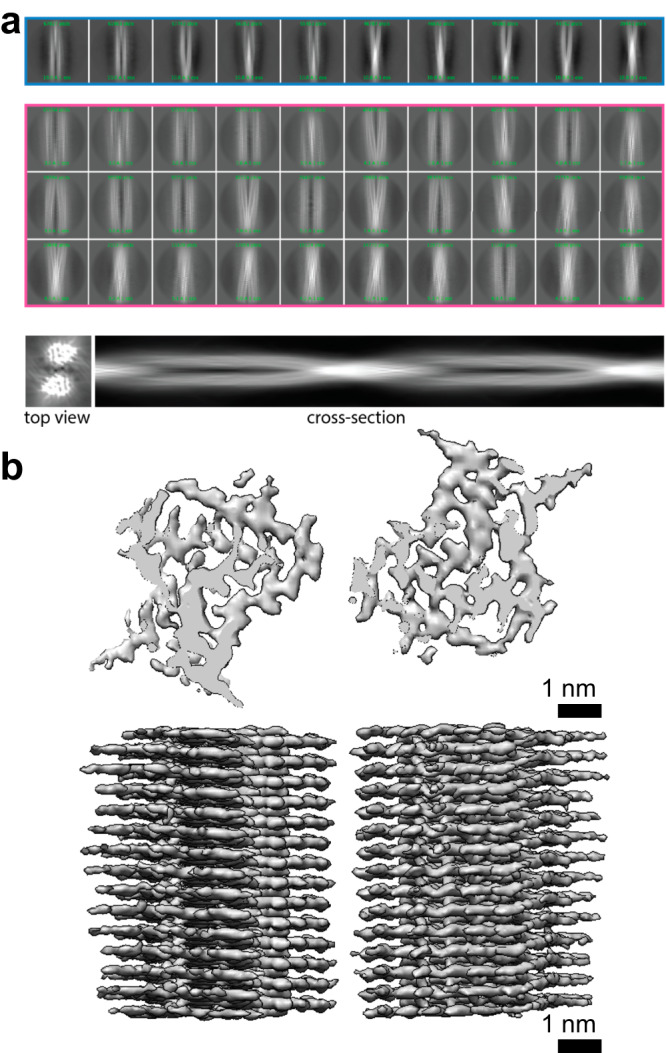


The correct version of Fig. 5 is:
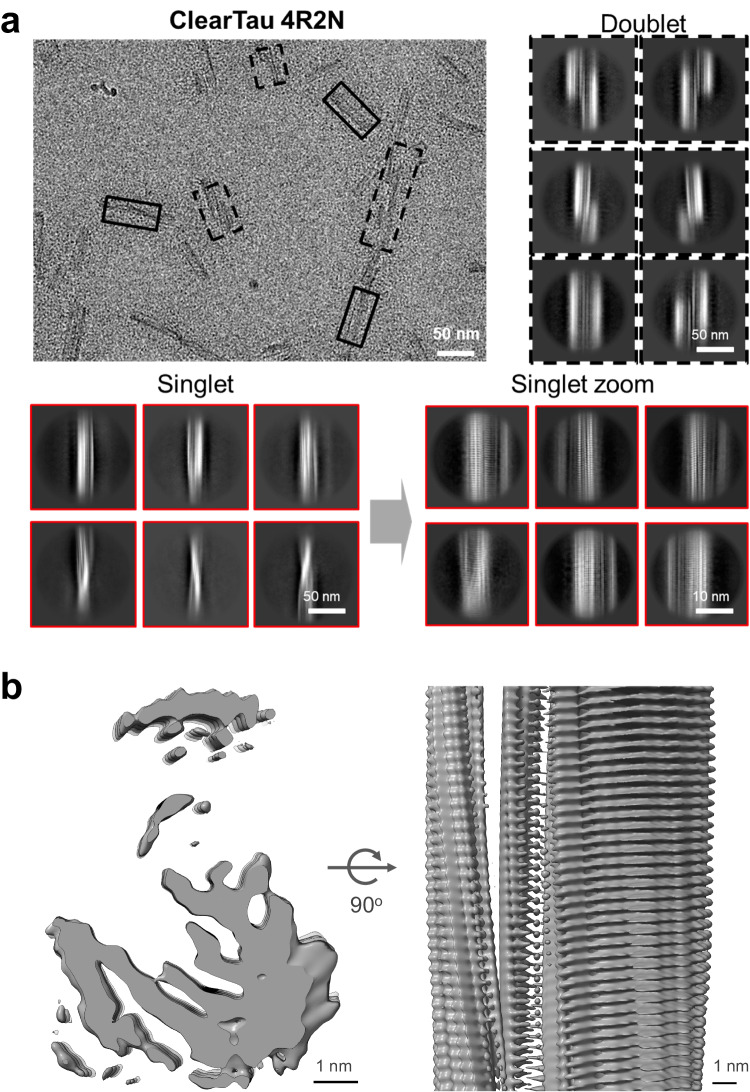


The original version of the Supplementary Information associated with this Article also contained an error in Supplementary Fig. 6 in which the wrong sample had been described. The HTML has been updated to include a corrected version of the Supplementary Information.

### Supplementary information

Updated Supplementary Information

