# Supplementary material for: Fully co-factor-free ClearTau platform produces seeding-competent Tau fibrils for reconstructing pathological Tau aggregates
Source: Nat Commun. 2023 Jul 4;14:3939. doi: 10.1038/s41467-023-39314-7 (PMC10319797; doi:10.1038/s41467-023-39314-7)
Supplement: Supplementary file 1 — Supplementary Information [file 41467_2023_39314_MOESM1_ESM.pdf]

Fully co-factor-free ClearTau platform produces seeding-competent Tau fibrils for reconstructing pathological Tau aggregates

Galina Limorenko[1], Meltem Tatli[2], Rajasekhar Kolla[1], Sergey Nazarov[4], Marie-Theres Weil[5], David C. Schöndorf[5], Daniela Geist[5], Peter Reinhardt[5], Dagmar E. Ehrnhoefer[5], Henning Stahlberg[2,3], Laura Gasparini[5] and Hilal A. Lashuel[1*]

[1] Laboratory of Molecular and Chemical Biology of Neurodegeneration, Institute of Bioengineering, School of Life Sciences, Ecole Polytechnique Fédérale de Lausanne, CH-1015 Lausanne, Switzerland.

[2] Laboratory of Biological Electron Microscopy, Institute of Physics, School of Basic Sciences, Ecole Polytechnique Fédérale de Lausanne

[3] Department of Fund. Microbiology, Faculty of Biology and Medicine, University of Lausanne, CH-1015 Lausanne, Switzerland

[4] Biological Electron Microscopy Facility, School of Life Sciences, Ecole Polytechnique Fédérale de Lausanne, CH-1015 Lausanne, Switzerland.

[5] Neuroscience Discovery, AbbVie Deutschland GmbH & Co KG, Knollstrasse, 67061 Ludwigshafen, Germany.

* email: hilal.lashuel@epfl.ch

Keywords: Tau, ClearTau, fibrils, aggregation, co-factor-free, heparin-free

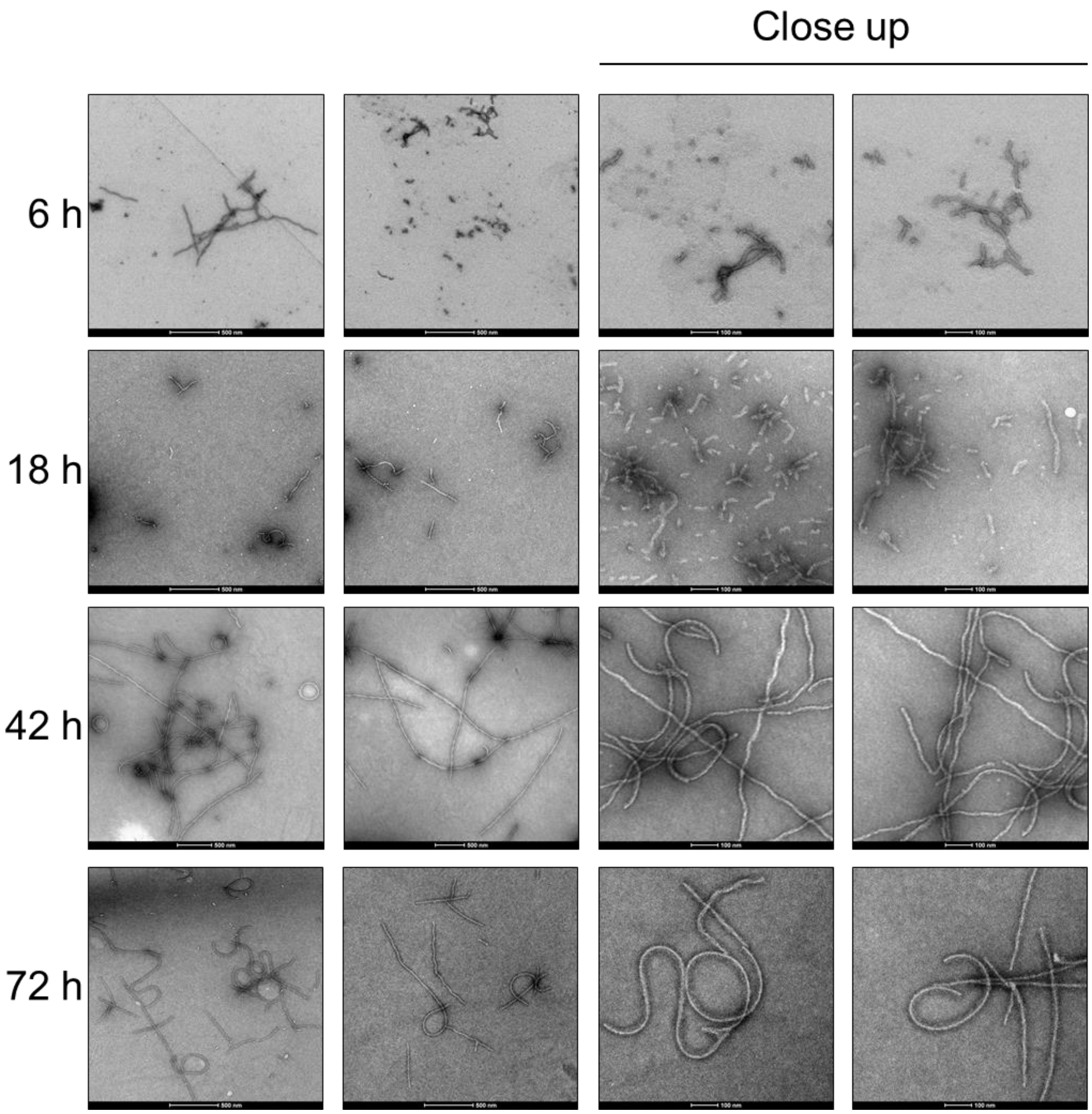

Supplementary Figure 1. Electron micrograph gallery of 4R2N ClearTau fibril aggregation overtime. The experiment was repeated of minimum three independent times with consistent results.

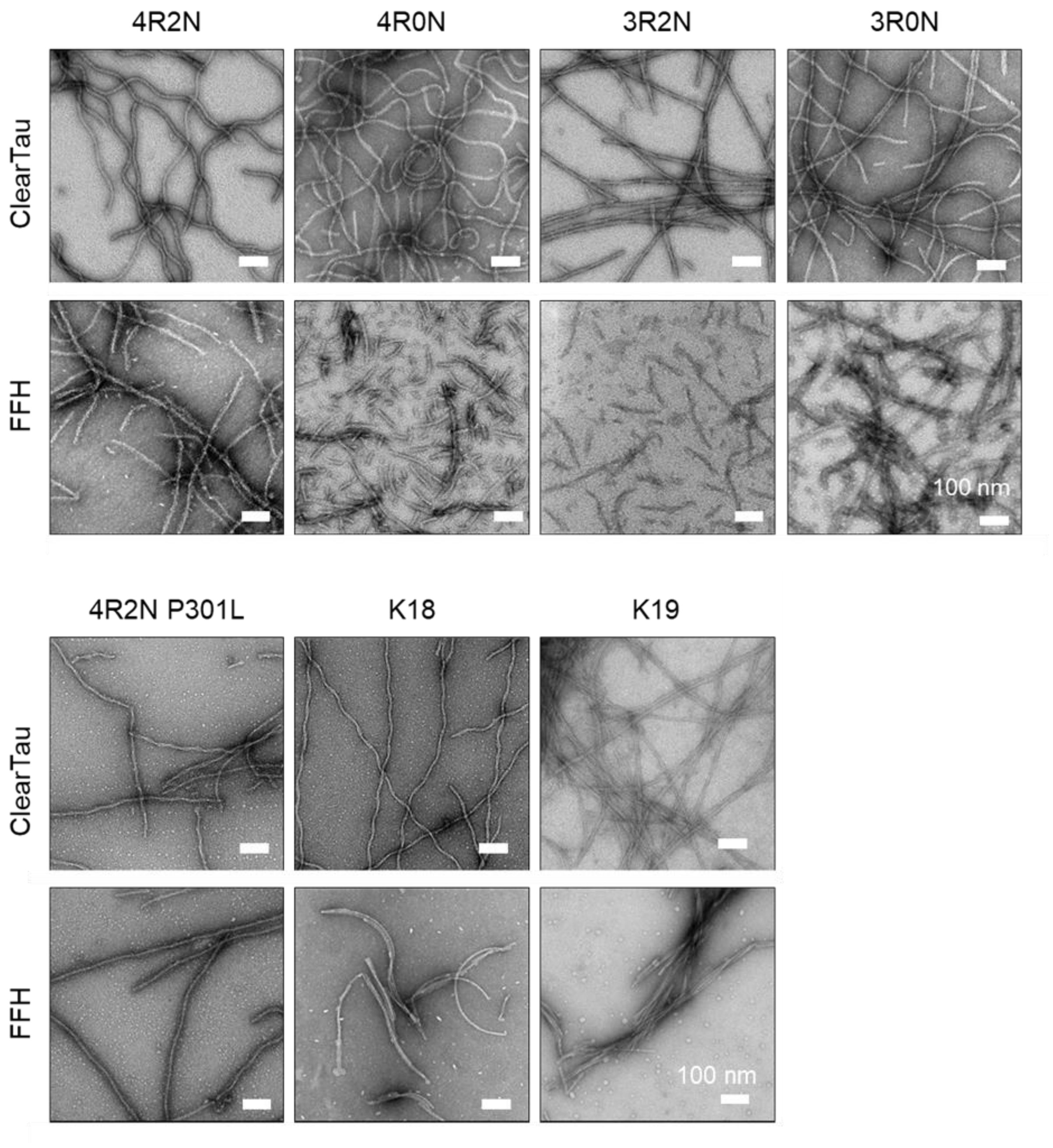

Supplementary Figure 2. A gallery of electron micrographs illustrating Tau variants' fibrillization by the ClearTau method and in the presence of FFH. The size bar = 100 nm. The experiment was repeated of minimum three independent times with consistent results.

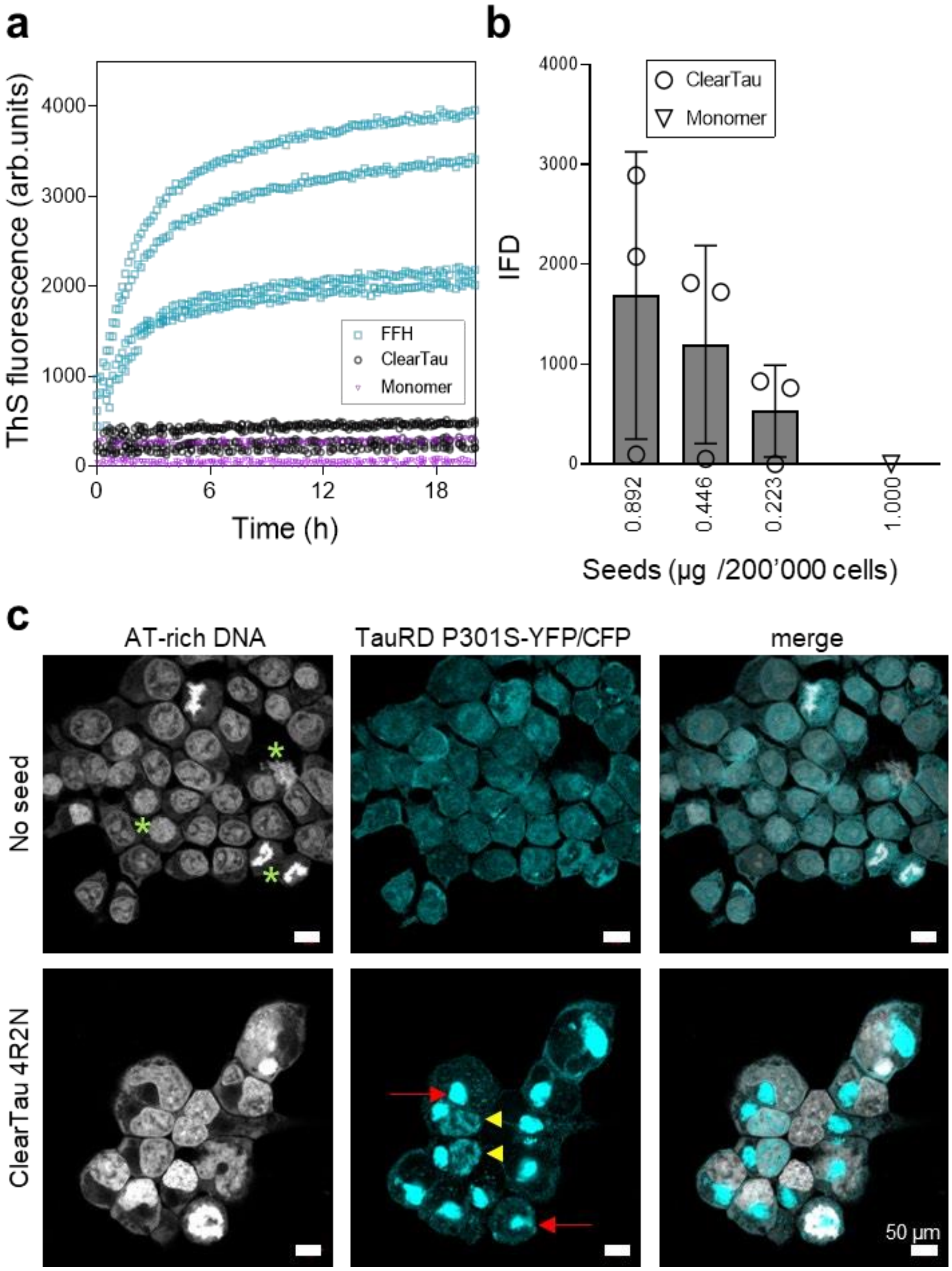

Supplementary Figure 3. ClearTau Tau preformed fibril seeding potency. a In vitro seeding of Tau 4R2N monomer with the ClearTau preformed fibrillar seed at 1:4 molar ratio (identical to heparin-to-Tau ratio). Data were collected in four independent experiments in triplicates, each for ClearTau seed and FFH, and four independent experiments in triplicates for monomer-only conditions. Data are presented as all individual values plotted. b FRET flow cytometry assessment of the HEK293T TauRDP301S biosensor cell line with added ClearTau 4R2N preformed fibrillar seed. IFD = integrated FRET density. Experiments were performed in triplicates, a minimum of 100,000 cells per run were sorted. Data are presented as mean values +/- SD with all individual values plotted. c Confocal imaging ClearTau 4R2N fibrillar seed assessment of cytoplasmic (red arrows) and nuclear reporter foci (yellow arrowheads) formation in HEK293T TauRD P301S biosensor cell line. Nuclei are demarcated by AT-rich DNA stain DRAQ5. Scale bars = 50 µm. The experiment was repeated of minimum three independent times with consistent results. Source data are provided as a Source Data file.

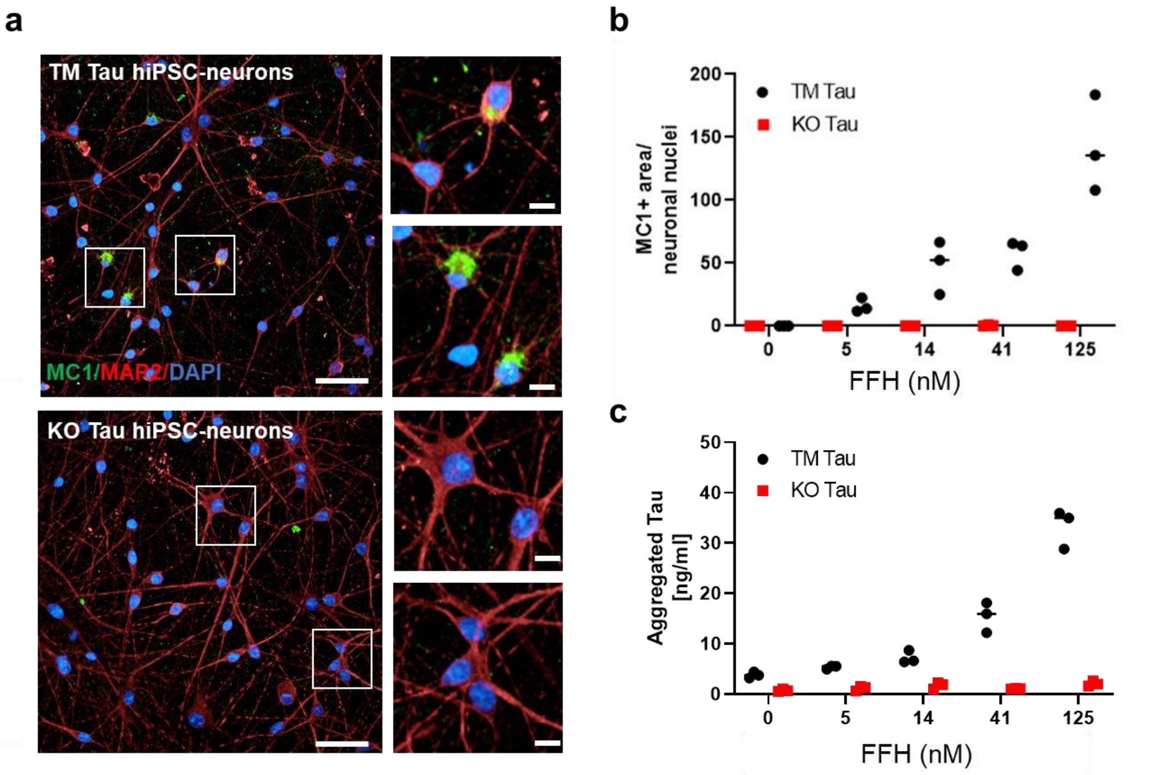

Supplementary Figure 4. FFH Tau fibrils induced dose-dependent aggregation of endogenous Tau in TM Tau, but not KO Tau, hiPSC-derived cortical neurons. TM Tau and TauKO hiPS-derived cortical neurons were exposed to different amounts of FFH Tau fibrils generated from recombinant P301L 2N4R Tau. 3 weeks later, the neurons were stained with the MC1 Tau antibody to detect endogenous tau aggregates (green) and MAP2 to stain the neurites (red). Nuclei were stained by DAPI. **a** Representative stack images of TM Tau and KO Tau hiPS-derived neurons treated with 125 nM FFH Tau. **b** Quantification of MC1+ area over neuronal nuclei. Scale bar 50 µm in main panels; 10 µm in insets. Two-way ANOVA: seed concentration $p < 0.0001$, genotype $p < 0.0001$. **c** Aggregation of endogenous Tau was evaluated by symmetric ELISA (Tau12/Tau12, N = 2 independent cultures, n = 3 replicates each). Two-way ANOVA: Tau concentration $p < 0.0001$, genotype $p < 0.0001$. Images and graphs represent data from one experiment. 2 independent experiments with 3 replicates/condition were performed for immunofluorescence analysis. In one experiment, biochemical analysis was also performed. Source data are provided as a Source Data file.

Supplementary Table 1. Quantification of ClearTau isoform fibril widths

	**4R2N**	**4R1N**	**4R0N**	**3R2N**	**3R1N**	**3R0N**
Number of fibrils	618	470	621	225	521	516
Median	15.17	15.69	15.67	14.49	14.05	12.35
Mean	15.20	15.83	15.70	14.73	14.49	12.49
Std. Deviation	2.24	3.49	3.04	2.55	3.19	2.59

Supplementary Table 2. Quantification of ClearTau isoform mixtures' fibril widths

	**All**			**2N +1N**			**2N**			**1N**		
ClearTau reaction	**1**	**2**	**3**	**1**	**2**	**3**	**1**	**2**	**3**	**1**	**2**	**3**
Number of fibrils	307	313	337	230	210	89	145	89	89	116	108	124
Mean	15.15	11.02	15.08	16.71	16.52	15.3	17.08	16.66	15.11	19.84	16.46	17.01
Std. Deviation	3.03	1.83	3.00	3.25	3.23	3.08	3.39	2.96	2.99	3.44	3.43	3.40

Supplementary Table 3. Quantification of ClearTau and FFH 4R2N P301L fibril widths pre- and post-proteolysis with proteinase K.

	**ClearTau 4R2N P301L**		**FFH 4R2N P301L**	
Reaction	**-PK**	**+PK**	**-PK**	**+PK**
Number of fibrils	200	200	200	200
Mean	18.50	15.10	12.90	9.50
Std. Deviation	2.50	2.23	2.84	1.85

Supplementary Table 4. Quantification of widths of Tau fibrils in the presence of compounds.

	**DMSO**	**ATPZ**	**Pyrocatechol violet**	**BSc3094**	**LMTX**	**Myricetin**	**Dopamine**	**L-DOPA**
Number of fibrils	100	58	103	60	101	104	102	101
Mean (nm)	15.0	13.3	9.15	14.4	14.6	9.45	20.7	11.3
Std. Deviation	3.23	2.34	1.72	2.65	2.38	1.70	2.79	2.72

Source data are provided as a Source Data file.

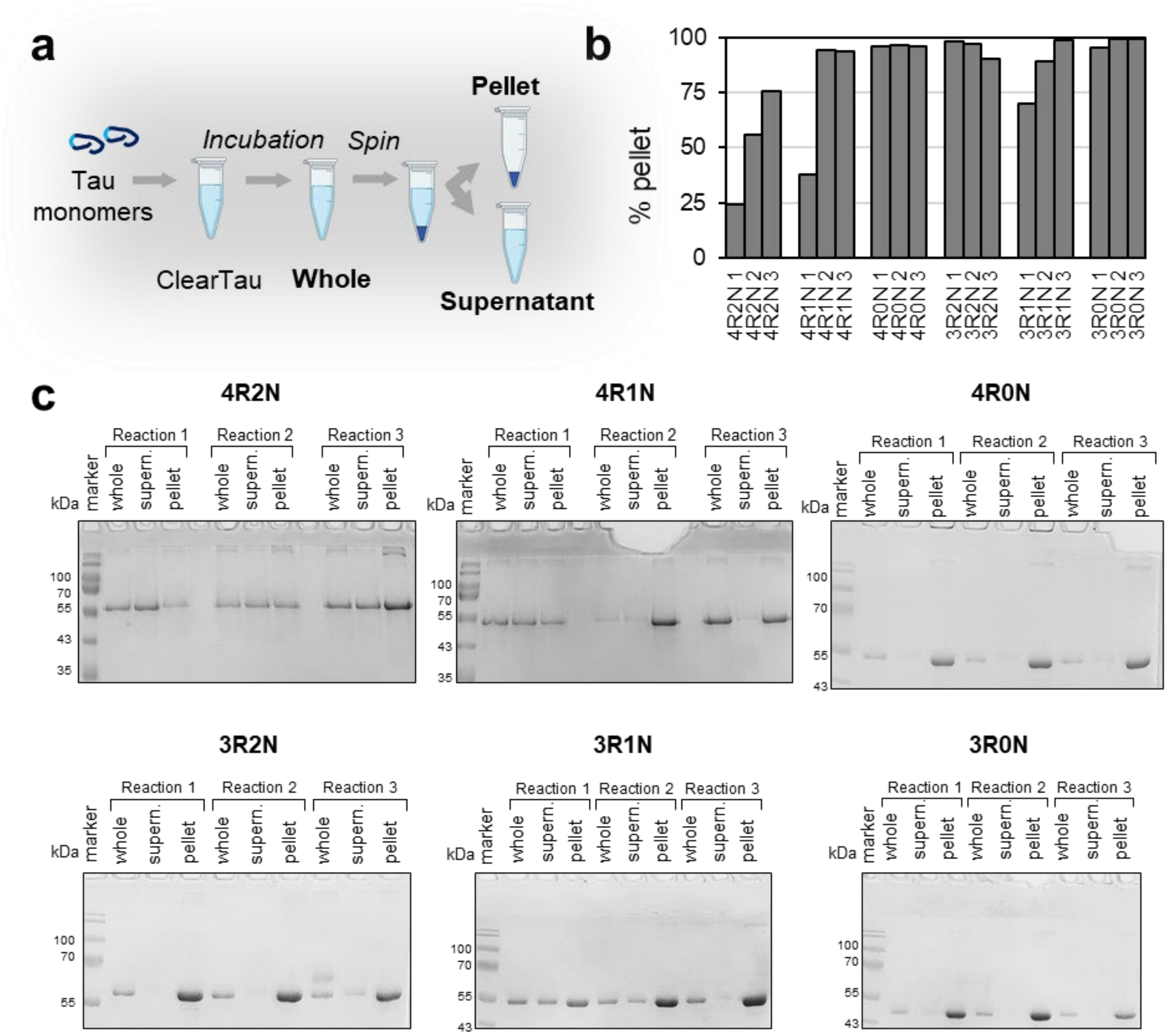

Supplementary Figure 5. Characterisation of monomer incorporation into the fibrilcontaining Tau pellet fraction across three independent ClearTau repeats for each Tau isoform. Three independent ClearTau method aggregation reactions for each Tau isoform (18 in total) were set up at 100 µM for 48 h at 37 °C under shaking conditions. The ~500 µl mixtures were ultracentrifuged at 100'000g for 1 h, supernatant was removed, pellets washed in dH_2O twice and resuspended in 500 ul of dH_2O. PAAG gels were run by loading 10 µl of supernatant or pellet + 10 µl 2X Laemmli buffer, or 5 µl Whole + 15 µl Laemmli buffer. Whole samples were loaded at half the amount to prevent the oversaturation. Whole sample signals were not included in the quantification. **a** Workflow of the fractionation protocol to separate the Tau fibrils (*pellet*) and monomers (*supernatant*). Created with BioRender.com. **b** Quantification of the monomer-to-fibril proportion of all 18 reactions shows efficient incorporation of monomers into the fibrillar fraction as early as at 48 h of the reaction in the current set up. **c** The whole, supernatant and pellet fractions were loaded on the SDS-PAGE gel and stained with the Coomassie total protein stain to assess fibrillization efficiency and monomer incorporation. All isoforms across all three independent repeats show efficient formation of the fibrils separated into the pellet fractions. Source data are provided as a Source Data file.

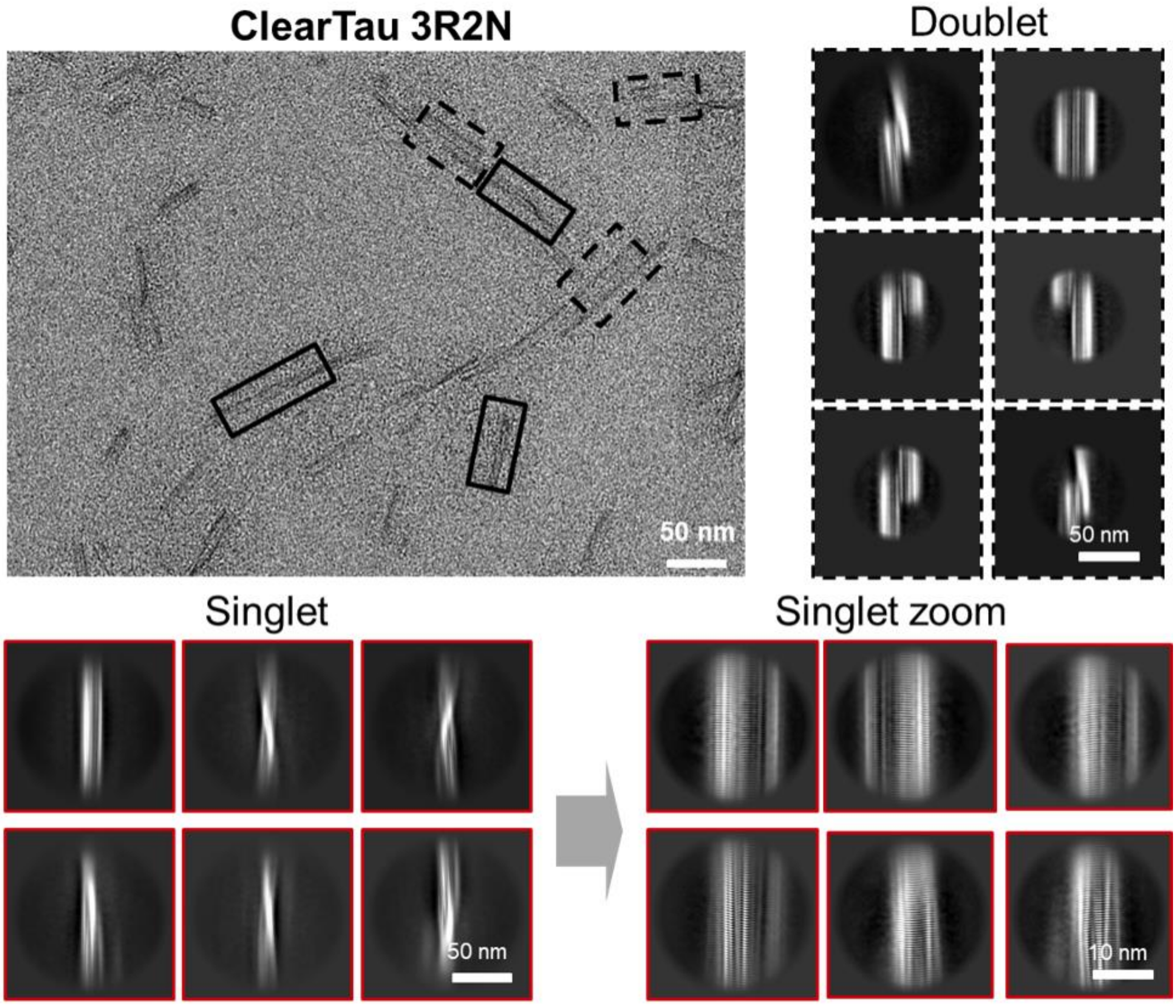

Supplementary Figure 6. Cryo-EM micrograph gallery of fibrils from ClearTau 3R2N. a ClearTau 3R2N. Selected singlets and doublets are outlined with red or yellow boxes. Representative 2D class averages of singlets and doublets from large 900-pixel segments downscaled to 300 pixels with a visible twist of the amyloid core. Representative 2D class averages of singlets from 300-pixel non-scaled segments with clear amyloid core and 4.77 Å separation of beta-strands. Scale bars = 10 and 50 nm.

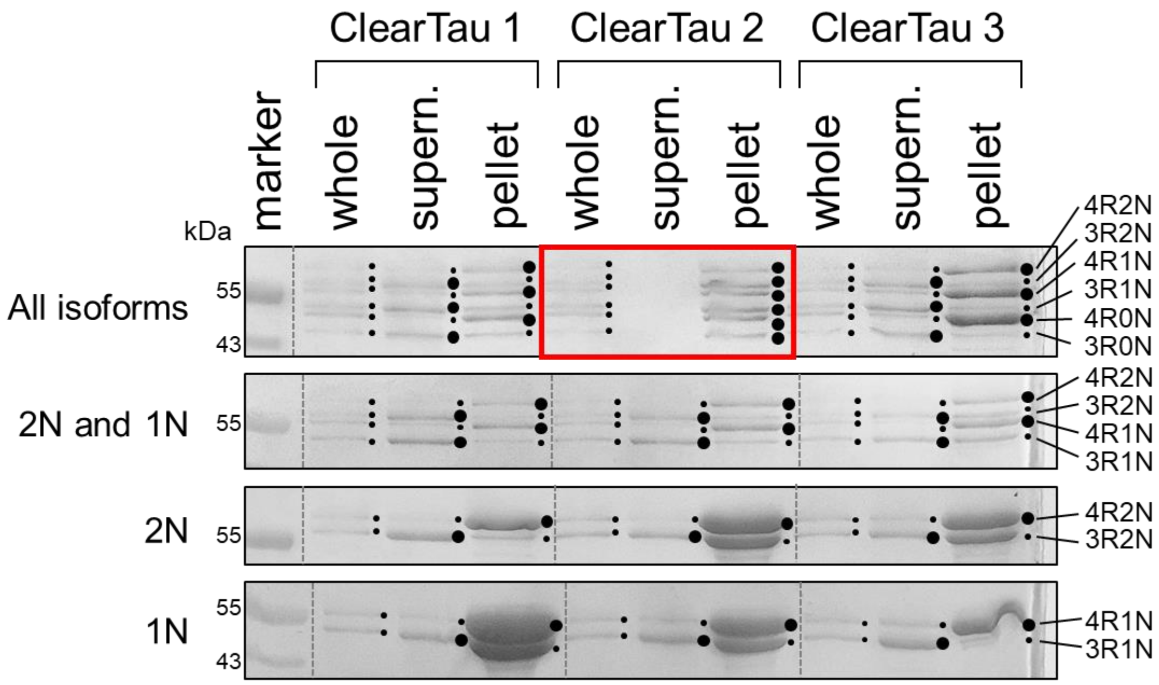

Supplementary Figure 7. Annotated SDS-PAGE gel of the Tau isoform mixtures shows relative incorporation of the Tau isoform monomers into the ClearTau fibrils. The circle size besides each band indicates the relative amount for better perception. The *whole* samples contain equal amounts of all isoforms as expected. Across the triplicate repeats for all mixtures, the 4R-containing Tau isoforms were more efficiently incorporated into the fibrils. The notable exception was All isoforms Sample 2 (red box), that demonstrated the complete incorporation of all the Tau monomers into the fibrils, at the same time showing the highest thioflavin S fluorescence and singlet Tau filaments (see main text). The ClearTau reactions were performed at 100 µM initial Tau concentration for 48 h with orbital shaking at 100 g at 37 °C. The ~500 µl mixtures were ultracentrifuged at 100’000 g for 1 h, supernatant was removed, pellets washed in dH_2O twice and resuspended in 500 µl of dH_2O. Polyacrylamide gels were run by loading 10 µl of supernatant or pellet + 10 µl 2X Laemmli buffer, or 5 µl Whole + 15 µl Laemmli buffer. Whole samples were loaded at half the amount to prevent the oversaturation. Source data are provided as a Source Data file.

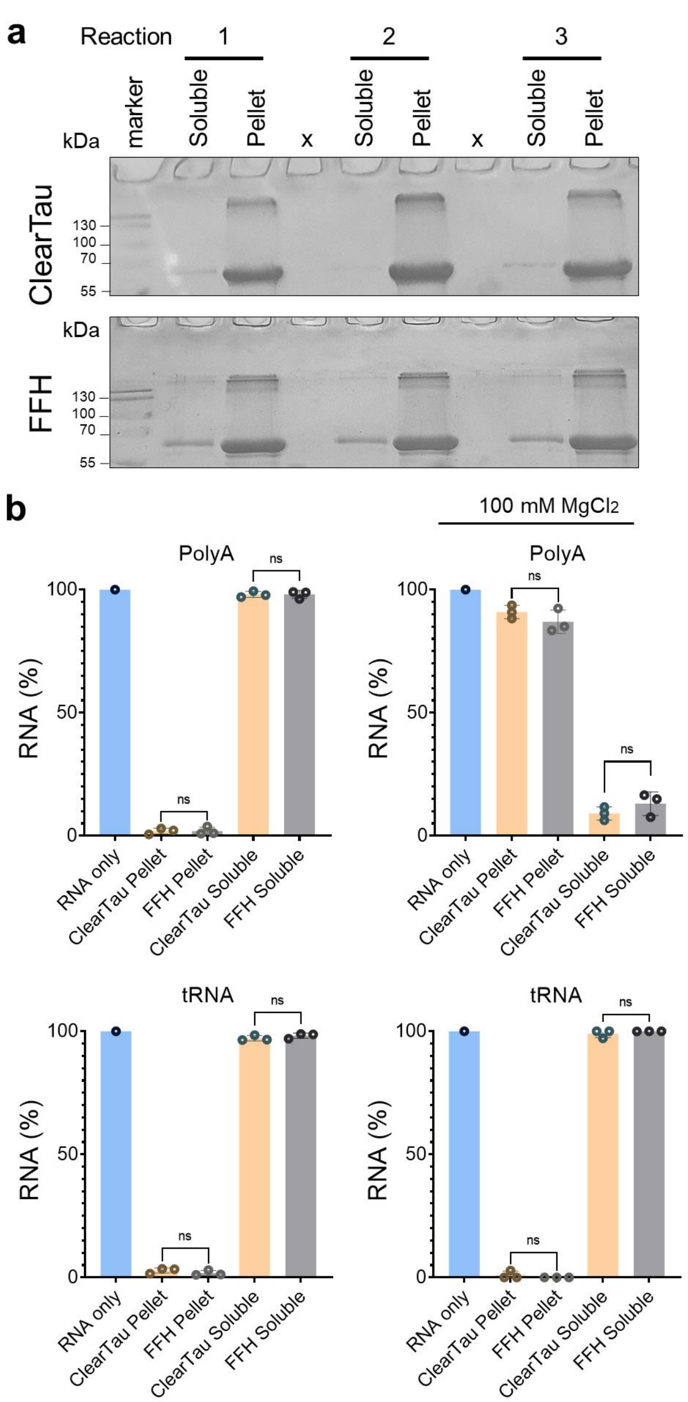

Supplementary Figure 8. Monomer incorporation into the fibrils and RNA-binding capacity of Tau 4R2N P301L prepared by the ClearTau method or in the presence of FFH. a Three independent samples were fibrillized at 100 µM for 24h, the ~500 µl mixtures were ultracentrifuged at 100'000g for 1 h, supernatant was removed, pellets washed in dH_2O twice and resuspended in 500 µl of dH_2O. Polyacrylamide gels were run by loading 10 µl of supernatant or pellet + 10 µl 2X Laemmli buffer. **b** PolyA and tRNA binding capacity of 4R2N P301L fibrils in the aggregation buffer and in the higher ionic strength buffer (+ 100 mM $MgCl_2$). N=3 biologically independent samples examined over 3 independent experiments. Data are presented as mean values +/- SD with all individual values plotted. Two-way ANOVA with Tukey's multiple comparisons test. Significant values are denoted by: ns P>0.05, * P≤0.05, ** P ≤0.01, *** P≤0.001, **** P≤0.0001. Exact p-values can be found in the Source Data file. Source data are provided as a Source Data file.

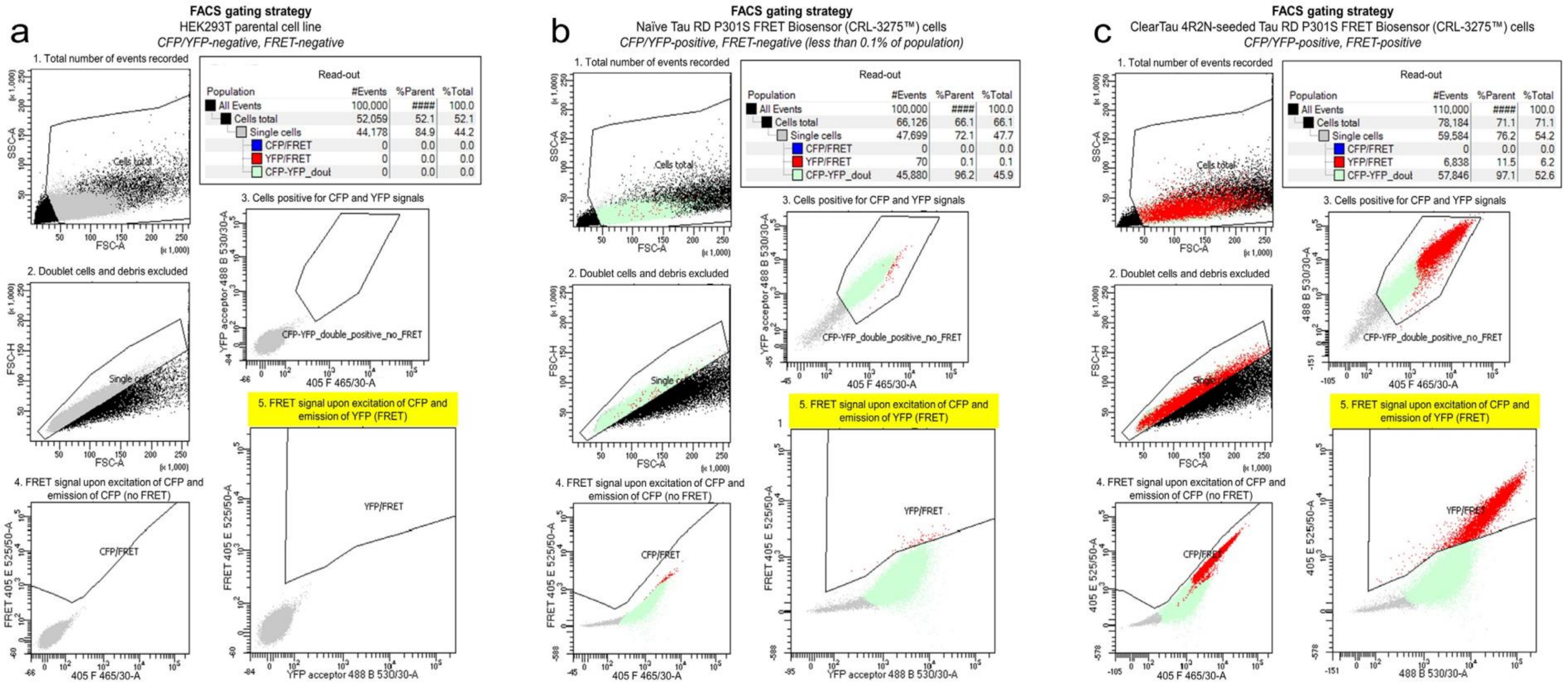

Supplementary Figure 9. FRET flow cytometry gating strategy. FRET vs. CFP donor bivariate plots depict the CFP/YFP-negative and FRETnegative (a) HEK293T parental cell line, CFP/YFP-positive and FRET-negative naïve Tau RD P301S FRET Biosensor (CRL-3275™) cells (b), and CFP/YFP-positive and FRET-positive ClearTau fibril-seeded Tau RD P301S FRET Biosensor (CRL-3275™) cells**.**

Supplementary References

1. Fitzpatrick, A. W. P. et al. Cryo-EM structures of tau filaments from Alzheimer's disease. Nature 547, 185-190, doi:10.1038/nature23002 (2017).
