# Supplementary material for: Fully co-factor-free ClearTau platform produces seeding-competent Tau fibrils for reconstructing pathological Tau aggregates
Source: Nat Commun. 2023 Jul 4;14:3939. doi: 10.1038/s41467-023-39314-7 (PMC10319797; doi:10.1038/s41467-023-39314-7)
Supplement: Supplementary file 2 — Reporting Summary [file 41467_2023_39314_MOESM2_ESM.pdf]

nature portfolio

Corresponding author(s): Hilal Lashuel

Last updated by author(s): May 16, 2023

Reporting Summary

Nature Portfolio wishes to improve the reproducibility of the work that we publish. This form provides structure for consistency and transparency in reporting. For further information on Nature Portfolio policies, see our Editorial Policies and the Editorial Policy Checklist.

Statistics

For all statistical analyses, confirm that the following items are present in the figure legend, table legend, main text, or Methods section.

n/a	Confirmed	
☐	☒	The exact sample size (*n*) for each experimental group/condition, given as a discrete number and unit of measurement
☐	☒	A statement on whether measurements were taken from distinct samples or whether the same sample was measured repeatedly
☐	☒	The statistical test(s) used AND whether they are one- or two-sided<br>*Only common tests should be described solely by name; describe more complex techniques in the Methods section.*
☐	☒	A description of all covariates tested
☐	☒	A description of any assumptions or corrections, such as tests of normality and adjustment for multiple comparisons
☐	☒	A full description of the statistical parameters including central tendency (e.g. means) or other basic estimates (e.g. regression coefficient) AND variation (e.g. standard deviation) or associated estimates of uncertainty (e.g. confidence intervals)
☒	☐	For null hypothesis testing, the test statistic (e.g. *F*, *t*, *r*) with confidence intervals, effect sizes, degrees of freedom and *P* value noted<br>*Give P values as exact values whenever suitable.*
☒	☐	For Bayesian analysis, information on the choice of priors and Markov chain Monte Carlo settings
☒	☐	For hierarchical and complex designs, identification of the appropriate level for tests and full reporting of outcomes
☒	☐	Estimates of effect sizes (e.g. Cohen's *d*, Pearson's *r*), indicating how they were calculated

Our web collection on statistics for biologists contains articles on many of the points above.

Software and code

Policy information about availability of computer code

Data collection	FLUOstar Omega microplate reader (BMG LABTECH, Germany) software, Tecnai Spirit BioTWIN transmission electron microscope software, BD LSR Fortessa software, Zeiss LSM 700 microscope software, Jasco J-815 CD spectrometer software, ThermoScientific 200kV Glacios software, Nanodrop One spectrophotometer software, Operetta CLS High-Content Analysis System (Perkin Elmer), Anthos LEDetect microplate reader (Anthos Mikrosysteme GmbH, Frisoythe, Germany). EMBD ID entry for ClearTau 4R2N is 16812 [https://www.ebi.ac.uk/pdbe/entry/emdb/EMD-16812], all data are verified and publicly available
Data analysis	ImageJ Measurement Tool (ImageJ, RRID:SCR_003070), MS Office suite, SerialEM, cryoSPARCv3.2, cryoSPARC, FOCUS, e2helixboxer.py from EMAN2, RELIONv3.1, GraphPad PRISM v9, Harmony Software (Perkin Elmer, version 4.9).

For manuscripts utilizing custom algorithms or software that are central to the research but not yet described in published literature, software must be made available to editors and reviewers. We strongly encourage code deposition in a community repository (e.g. GitHub). See the Nature Portfolio guidelines for submitting code & software for further information.

Data

Policy information about availability of data

All manuscripts must include a data availability statement. This statement should provide the following information, where applicable:

- Accession codes, unique identifiers, or web links for publicly available datasets
- A description of any restrictions on data availability
- For clinical datasets or third party data, please ensure that the statement adheres to our policy

Source data are provided with this paper in the Source Data file. CryoEM structure EMD ID:16812.

Human research participants

Policy information about studies involving human research participants and Sex and Gender in Research.

Reporting on sex and gender	N/A
Population characteristics	N/A
Recruitment	N/A
Ethics oversight	N/A

Note that full information on the approval of the study protocol must also be provided in the manuscript.

Field-specific reporting

Please select the one below that is the best fit for your research. If you are not sure, read the appropriate sections before making your selection.

☒ Life sciences ☐ Behavioural & social sciences ☐ Ecological, evolutionary & environmental sciences

For a reference copy of the document with all sections, see nature.com/documents/nr-reporting-summary-flat.pdf

Life sciences study design

All studies must disclose on these points even when the disclosure is negative.

Sample size	Minimum three independent samples were used for each experiment with minimum of three technical replicates
Data exclusions	No data were excluded
Replication	All replicates were successful
Randomization	No experiments requiring randomization were conducted
Blinding	No experiments requiring blinding were conducted

Reporting for specific materials, systems and methods

We require information from authors about some types of materials, experimental systems and methods used in many studies. Here, indicate whether each material, system or method listed is relevant to your study. If you are not sure if a list item applies to your research, read the appropriate section before selecting a response.

Materials & experimental systems

n/a	Involved in the study
☐	☒ Antibodies
☐	☒ Eukaryotic cell lines
☒	☐ Palaeontology and archaeology
☒	☐ Animals and other organisms
☒	☐ Clinical data
☒	☐ Dual use research of concern

Methods

n/a	Involved in the study
☒	☐ ChIP-seq
☐	☒ Flow cytometry
☒	☐ MRI-based neuroimaging

Antibodies

Antibodies used

Secondary Alexa fluor 488-conjugated goat anti-mouse (Cat# A11001, Thermo Scientific, 1:500) and Alexa fluor 647-conjugated rabbit anti-chicken antibodies (Cat# 703-605-155, Jackson Laboratory,1:500) , primary antibodies were used: monoclonal mouse MC1 antibody (kindly provided by Peter Davies to AbbVie through a Material Transfer Agreement with Feinstein Institute; 2 μg/mL dilution) to detect the insoluble tau aggregates and anti-MAP2 antibody (chicken, Abcam, #5392; 1:5000 dilution) to counterstain the neurites, nuclei were counterstained by DAPI.

Validation

The validation of antibodies are available on the vendor websites Thermo Fisher, Jackson Laboratory, Abcam. MC1 antibody is described here: Jicha GA, Bowser R, Kazam IG, Davies P. Alz-50 and MC-1, a new monoclonal antibody raised to paired helical filaments, recognize conformational epitopes on recombinant tau. J Neurosci Res. 1997 Apr 15;48(2):128-32. doi: 10.1002/(sici)1097-4547(19970415)48:2<128::aid-jnr5>3.0.co;2-e. PMID: 9130141.

Eukaryotic cell lines

Policy information about cell lines and Sex and Gender in Research

Cell line source(s)

ATCC®, Sigma

Authentication

HEK293T CRL-3275™ Tau RD P301S FRET Biosensor, iPSC0028

Mycoplasma contamination

Cell lines not tested for mycoplasma contamination after purchase from vendors

Commonly misidentified lines
(See ICLAC register)

N/A

Flow Cytometry

Plots

Confirm that:

☒ The axis labels state the marker and fluorochrome used (e.g. CD4-FITC).

☒ The axis scales are clearly visible. Include numbers along axes only for bottom left plot of group (a 'group' is an analysis of identical markers).

☒ All plots are contour plots with outliers or pseudocolor plots.

☒ A numerical value for number of cells or percentage (with statistics) is provided.

Methodology

Sample preparation

Cell line Tau RD P301S FRET Biosensor (CRL-3275™) was acquired from ATCC® and maintained in DMEM medium with 0.5 % L-Glutamine, 0.5 % penicillin-streptomycin antibiotic cocktail and 10 % foetal bovine serum supplementation (Gibco, Thermo Fisher Scientific). BS cells were plated in poly-L-lysine treated 6 well plates at a density of 100'000 cell/well. Cells were allowed to grow and divide in an incubator at 37 °C. BS cultures were allowed to reach a confluency of 50-60%. The ClearTau sonicated fibril seeds were incubated with Lipofectamine2000 at 1:2 ratio weight to volume in OptiMEM (Gibco, Thermo Fisher Scientific). The cultures were transduced with ClearTau sonicated fibril seeds at amounts of 0.892 μg, 0.446 μg and 0.223 μg of fibrils per 200'000 cells. Cells were exposed to the fibrils for 4 h, cultures were washed twice in phosphate-buffered saline (PBS, Gibco, Thermo Fisher Scientific) to remove all residual seeds, and further incubated for 96 h in standard medium to allow to have two cell division cycles. Cultures were washed once in PBS, and dissociated using 200 μl Trypsin-EDTA 0.05% for 5 min at 37 °C. 150 μl of DMEM medium was added to each well to neutralize Trypsin action, cells were gently dissociated into single cells by pipetting, they were transferred into Eppendorf tubes and centrifuged at 1000g at room temperature for 5 min. The supernatant was removed, and cells were re-suspended in the 900 μl of 2% paraformaldehyde (Thermo Fisher Scientific) and incubated for 10 min, then pelleted by centrifugation at 1000 g 4 °C for 5 min. The supernatant was removed, and the pellet was re-suspended in HBSS.

Instrument

FRET detection was performed using BD LSR Fortessa with excitation-emission laser filters: 405 – 465/30, 488 – 530/30, with FRET signal detectable at 405 – 530/30 couple

Software	FRET detection was performed using BD LSR Fortessa with excitation-emission laser filters: 405 – 465/30, 488 – 530/30, with FRET signal detectable at 405 – 530/30 couple. Parental HEK293T cells were used to define the cell population on the SC-A vs FCS-A bivariate plot. Doublet events were excluded on FSC-H vs. FSC-A bivariate plot. Voltages were adjusted to exclude any signal on CFP, YFP, or FRET filters. Double-positive CFP-YFP BS cell population was defined by the negative Lipofectamine-only BS cell line control sample. Compensation was adjusted to remove any bleed-through of CFP and YFP signal to the FRET channel.<br>For data analysis in Fortessa software cell populations were gated to exclude the debris events and doublets. Negative control (BS-Lipofectamine) was used to define double-positive cell population, spill-over to the FRET channel was excluded on CFP-FRET and YFP-FRET bivariate plots. For each sample, percent of FRET-positive events and Median of fluorescence intensity were recorded, and the product was plotted to represent Integrated FRET Density (IFD). Three independent experiments were performed for each condition with a minimum of 100’000 events per sample recorded. The plot represents average measurements; bars represent standard deviation. Figure plotting was made in MS Office tools suite.
Cell population abundance	Minimum 100'000 cells per sample
Gating strategy	For data analysis in Fortessa software cell populations were gated to exclude the debris events and doublets. Negative control (BS-Lipofectamine) was used to define double-positive cell population, spill-over to the FRET channel was excluded on CFP-FRET and YFP-FRET bivariate plots. For each sample, percent of FRET-positive events and Median of fluorescence intensity were recorded, and the product was plotted to represent Integrated FRET Density (IFD).

☒ Tick this box to confirm that a figure exemplifying the gating strategy is provided in the Supplementary Information.
